# Development and Validation of a Questionnaire to Measure Chinese Preschool Teachers’ Implementation of Social-Emotional Practices

**DOI:** 10.3389/fpsyg.2021.699334

**Published:** 2021-09-10

**Authors:** Li Luo, Patricia Snyder, Yuxi Qiu, Anne Corinne Huggins-Manley, Xiumin Hong

**Affiliations:** ^1^College of Preschool Education, Capital Normal University, Beijing, China; ^2^College of Education, University of Florida, Gainesville, FL, United States; ^3^College of Arts, Sciences and Education, Florida International University, Miami, FL, United States; ^4^Faculty of Education, Beijing Normal University, Beijing, China

**Keywords:** social-emotional practices, preschool teachers, measurement, psychometric properties, mainland China

## Abstract

We describe the development and validation of the *Social-Emotional Teaching Practices Questionnaire-Chinese* (SETP-C), a self-report instrument designed to gather information about Chinese preschool teachers’ implementation of social-emotional practices. Initially (study 1), 262 items for the SETP-C were generated. Content validation of these items was conducted separately with Chinese practice experts, research experts, and preschool teachers. Significant revisions were made to items based on theoretical evidence and empirical findings from initial content validation activities, which led to a 70-item version of the SETP-C. In study 2, preliminary psychometric integrity evidence and item characteristics of the SETP-C were gathered based on the data from a sample of 1,599 Chinese preschool teacher respondents. Results from confirmatory factor analyses suggested a seven-factor measurement model, and high internal consistency score reliability was documented for each dimension of the SETP-C. Results of item response theory graded response models further indicated adequate psychometric properties at the item level.

## Introduction

Social-emotional competence (SEC) refers to a child’s ability to form close and secure relationships with others; experience, regulate, and express emotions in socially and culturally appropriate ways; and explore the environment in appropriate ways to learn (Yates et al., 2008). A body of correlational and longitudinal research indicates that SEC of young children is positively related to their readiness for school and early school adjustment, whereas negatively related to a variety of later academic and behavioral problems (e.g., McClelland and Morrison, 2003; Trentacosta and Izard, 2007). Recent years have witnessed increased attention to the promotion of young children’s SEC, including in preschool programs. For example, a multitude of preschool curricula or programs have been designed and implemented to teach and foster SEC (e.g., Domitrovich et al., 2004; Hemmeter et al., 2016). To support young children to acquire SEC, teachers often use intentional and systematic teaching practices (Epstein, 2009). The value of instruction in SEC in early childhood educational settings has been documented both theoretically and empirically (e.g., Zins and Elias, 2006; Durlak et al., 2011). In mainland China, there has been growing interest in promoting the SEC of young children, and the social development domain has officially been identified as one of the five curricular domains in preschools nationwide since 2001 (Li and Feng, 2013).

One recent direction in the literature has been the use of teacher self-report instruments to explore which theoretically or empirically supported practices Chinese preschool teachers report they use to support children’s SEC (e.g., Luo et al., 2020; Ye and Sidhu, 2020). When combined with direct observations, this information is useful for improving practice implementation (Durlak and DuPre, 2008) and informing professional learning experiences for teachers (Luo et al., 2017; Hemmeter et al., 2018). However, there exists a lack of valid and reliable measurement instruments. For example, using an author-developed instrument, Ye (2012) surveyed preschool teachers from four provinces to investigate their attitudes toward, implementation of, and challenges in social education. Ye found that social education has been somewhat neglected during instruction and teachers were not well equipped to implement social education. One issue in that study was neither the instrument development process nor the psychometric properties of the instrument were described. This somewhat limits the validity of inferences that can be derived from the reported findings. In addition, only general information about preschool social-emotional instruction was gathered from teachers (e.g., whether and when they implemented a social-emotional lesson, objectives for the social-emotional lesson, and instructional approaches to teach SEC). The author did not examine the specific practices that preschool teachers were using to promote young children’s SEC.

The present study was designed to address the ongoing need for psychometrically defensible practice-focused instruments based on the SEC theoretical and empirical literature. These instruments would assist in characterizing and quantifying the practices of Chinese preschool teachers to foster young children’s SEC. The aim of the present investigation was to systematically develop and validate a measure of social-emotional practices for Chinese preschool teachers.

## Conceptual Frameworks

### National Requirements of Preschool Social Education in Mainland China

To improve the quality of preschools, the Ministry of Education of the People’s Republic of China (MOE) issued two milestone documents in preschool education, the *Guidelines for Preschool Education – Trial Version* (GPE) and the *Early Learning and Development Guidelines for Children Aged 3–6 Years* (ELDG). Issued in 2001 and widely considered the most influential source on teaching practices used in preschools nationwide, the GPE organized preschool curriculum into five domains: health, language, social development, sciences, and arts (Ministry of Education of the People’s Republic of China, 2001). For each curricular domain, the GPE describes the knowledge, competencies, and associated skills that Chinese teachers should have in order to promote children’s optimal learning and development within the context of preschool programs (Zhu, 2009). In 2012, the ELDG further divided the social development domain into two content areas: interpersonal relationships and social adaptability. Each content area is defined through one or more developmental goals that are then operationalized into behavioral markers. The behavioral markers are specified for three age bands (3-4, 4–5, and 5–6 years old) that correspond to the typical age groups of Chinese preschool classrooms (Ministry of Education of the People’s Republic of China, 2012).

Age-appropriate goals for preschool children’s development and learning and recommended educational practices outlined in these two Chinese landmark documents provide information about social-emotional practices that are socio-culturally valued and expected to be implemented by preschool teachers in mainland China.

### Teaching Pyramid Observation Tool for Preschool Classrooms

In the United States, the *Pyramid Model* for Promoting Social-Emotional Competence in Infants and Young Children (Fox et al., 2010; Hemmeter et al., 2016, 2021) provides a multi-tiered framework for organizing evidence-based environmental, interactional, and instructional practices to foster children’s SEC in early childhood classrooms. The *Pyramid Model* includes universal teaching practices to support the SEC of all children (Tier 1: universal promotion), the provision of targeted preventive social-emotional supports for children with or at-risk for social-emotional delays (Tier 2: secondary prevention), and individualized positive behavior supports for children with significant or persistent challenging behavior (Tier 3: tertiary intervention). The Teaching Pyramid Observation Tool for Preschool Classrooms (TPOT; Hemmeter et al., 2014) is an instrument designed to measure classroom-wide implementation of universal and targeted teaching practices associated with the *Pyramid Model* and the teacher’s capacity to individualize teaching practices and implement individualized behavior support plans. The TPOT is completed based on a combination of at least a 2-h observation in a preschool classroom and a 15- to 20-min interview with the teacher. The TPOT includes a total of 132 teaching practice indicators organized under three subscales: Key Practices, Red Flags, and Response to Challenging Behavior.

The effects of *Pyramid Model* practices on improving children’s SEC have been empirically supported (e.g., Hemmeter et al., 2016, 2021). Although most of the studies focused on the *Pyramid Model* have been conducted in the United States, in recent years, the *Pyramid Model* is receiving more attention internationally. The *Pyramid Model* has been documented in the Chinese professional literature and has been viewed as valuable to guide social-emotional practices (e.g., He and Zhang, 2014). Descriptive studies conducted in South Korea, Turkey, and mainland China have shown that without professional development support, preschool teachers were observed to implement or reported low levels of implementation of the *Pyramid Model* practices as measured by the TPOT or author-developed measures (e.g., Heo et al., 2014; Luo et al., 2017; Rakap et al., 2018). As for the TPOT, findings from a pilot study suggest that it appears feasible to administer this instrument in Chinese preschools, although some TPOT items/indicators need to be adapted to align better with Chinese culture, such as developing intensive and individualized interventions for children with persistent challenging behavior at the tertiary tier of the *Pyramid Model* (Luo et al., 2017).

### The Current Research

Given the lack of measure of Chinese preschool teachers’ social-emotional practices with adequate psychometric properties, we developed and validated the *Social-Emotional Teaching Practices Questionnaire-Chinese* (SETP-C), using the universal and secondary tiers of the *Pyramid Model* as an initial conceptual framework and being informed by the content of the Chinese guidelines previously described (see Supplementary Figure 1). The SETP-C was designed as a self-report instrument to measure Chinese preschool teachers’ frequency of and confidence with implementing social-emotional practices shown to promote young children’s SEC. As elaborated in the following sections, instrument development and validation occurred as part of two studies: development and content validation of the SETP-C (study 1) and preliminary psychometric analyses of the SETP-C scores using data obtained from a sample of preschool teacher respondents (study 2).

## Study 1: Development and Content Validation of the SETP-C

Following DeVellis (2012) guidelines for instrument development, we developed and refined the content of the SETP-C and documented validity evidence of content and response process in four phases. Supplementary Figure 2 depicts these four phases. Methods for each phase are discussed below. Institutional review board approval was obtained from the authors’ university. The SETP-C was initially written in English and then was translated into simplified Chinese by two translators independently. Their translations were compared and contrasted by two blind judges. Then, a fifth person confirmed the translations and processes. All individuals involved in these steps held a master’s or doctoral degree in early childhood education or comparative education field. The SETP-C was reverse translated into English by three doctoral students majoring in early childhood education to verify the accuracy of the translation.

### Phase 1: Item Generation and Selection

With the subject-centered approach (Crocker and Algina, 1986), SETP-C scores were intended to locate Chinese teachers at different points on a quantitative continuum with respect to their implementation of social-emotional practices. As the underlying construct, social-emotional practices were defined as specific actions or behaviors of preschool teachers that involve manipulating the physical, temporal, interactional, or instructional environment to foster young children’s SEC. Initial items for the SETP-C were generated with the goal of developing a comprehensive set of items that measure the social-emotional practices aligned with the *Pyramid Model* and two Chinese early learning and education standards documents (i.e., GPE and ELDG).

#### Examining Alignment

We conducted a crosswalk of the *Pyramid Model* practices as measured by the pre-publication version of the TPOT (Hemmeter et al., 2008) with practices stipulated in China’s GPE and ELDG documents, which demonstrated strong alignment and also strengthened the rationale for using the *Pyramid Model* as the conceptual framework to measure Chinese teachers’ implementation of social-emotional practices. The crosswalk involved the first author comparing teaching practices across the different resources and indicating where they converged. Our research team confirmed the alignment and when discrepancies occurred, they were resolved through discussion and reaching consensus about alignment. This alignment activity supported content-oriented validation evidence.

#### Field Observation

A small pilot study was conducted to explore the extent to which a sample of Chinese preschool teachers was implementing the social-emotional practices associated with the *Pyramid Model* and how congruent these practices were with the Chinese socio-cultural contexts. The pre-publication version of the TPOT was administered in 20 Chinese preschool classrooms in Beijing. Each teaching practice indicator on the TPOT was analyzed for item difficulty. Generally, teaching practices of medium difficulty (between 30 and 80%; Miller et al., 2013) were selected for further adaptation and content validation on the next iteration of the SETP-C to maximize the variance of future respondents’ scores.

#### Review of the Chinese Empirical Literature

Simultaneous to alignment and field observation activities, a systematic review of the Chinese literature was conducted in nine common English and Chinese educational electronic databases. A total of 76 published journal articles dating back to 1986 were included in the review. This systematic review was intended to identify and summarize teaching practices for promoting SEC of preschool children that have been empirically studied in Chinese preschools, with an emphasis on the identification of the four key components of instruction as described by Snyder et al. (2013): what to teach, when to teach, how to teach, and how to evaluate. Furthermore, an effort was made to review and pool items from published instruments that were designed to measure Chinese preschool teachers’ social-emotional instruction. However, only one relevant author-development instrument was identified (i.e., Ye, 2012). As described previously, neither the instrument development process nor the psychometric properties of that instrument were reported in Ye’s study.

#### Results

Building on the activities described above, we generated a list of 262 potential items. Given the content of these potential items were all related to teaching practices for promoting SEC or addressing challenging behavior of preschool children, a significant number of overlapping items were present in the item pool. These potential items were sorted according to the tier of *Pyramid Model* practices (i.e., universal promotion tier, secondary prevention tier) that they represented, with the intent to select key items from each tier classified in the conceptual framework of the SETP-C. To avoid redundancy, similar or repetitive items were identified between and within each tier and then redundant items were removed, combined, or reworded. Based on the psychometric analyses of items using data collected during the pilot study and a thorough review of items, 89 items were generated for the preliminary version of the SETP-C.

### Phase 2: Content Validation With Chinese Practice Experts

We conducted a content validation activity to examine Chinese practice experts’ perspectives about the importance and appropriateness of the 89 social-emotional practices included in the preliminary version of the SETP-C. In accordance with China’s *Preschool Principal Qualifications* (Ministry of Education of the People’s Republic of China, 2015), preschool principals are identified as practice experts who play an important role in preschool curriculum decision-making and in providing support and guidance to preschool teachers about their instruction and interactions with children.

#### Participants and Measure

A total of 256 Chinese practice experts attending a series of training workshops at China’s National Training Center for Preschool Principals were invited to complete a content validation rating scale for SETP-C items. These practice experts came from 31 provinces, autonomous regions, or direct-controlled municipalities in mainland China, and had been recommended to attend training workshops by their provincial Department of Education as prominent preschool principals. These practice experts were asked to rate each item on the preliminary version of the SETP-C for cultural relevance and importance, respectively on a 6-point ordinal scale, where 1 indicated that the item was *not at all relevant/important* and 6 indicated that the item was *extremely relevant/important*. The response rate was 83.2%. As shown in Supplementary Table 1, these practice experts were from different preschools and they identified themselves as either preschool principals (73.6%), vice principals (19.4%), or lead teachers (7.0%). About 84% of practice experts reported holding a bachelor’s or higher degree. Their average number of years of professional experience in preschool settings was 17.1 years, with a range from 1 to 47 years.

#### Data Analytic Procedures

Item analyses were conducted to examine mean, standard deviation, variance, and item discrimination for each item. Supplementary Tables 2, 3 show the descriptive statistics and score distributions on the *How Important* section and the *How Culturally Relevant* section for the 89 items. Principal components analyses were performed to investigate the underlying linear dimensions of the 89-item content validation rating scale using the data from the *How Culturally Relevant* section and the *How Important* section, respectively. To achieve parsimony and simple structure and to facilitate interpretation, the components were rotated to orthogonal structure using varimax rotation. A five-factor solution was supported using multiple criteria across extraction methods to produce dimensions that (a) satisfy Cattell’s scree test (Cattell, 1966), (b) retain five or more items with salient structure coefficient, (c) yield reasonable internal consistency score reliability, (d) have eigenvalues greater than 1.0 by Kaiser-Guttman rule (Brown, 2006), and (e) are interpretable in terms of parsimonious converge of the construct.

#### Item Reduction Procedures

**FIGURE 1:**
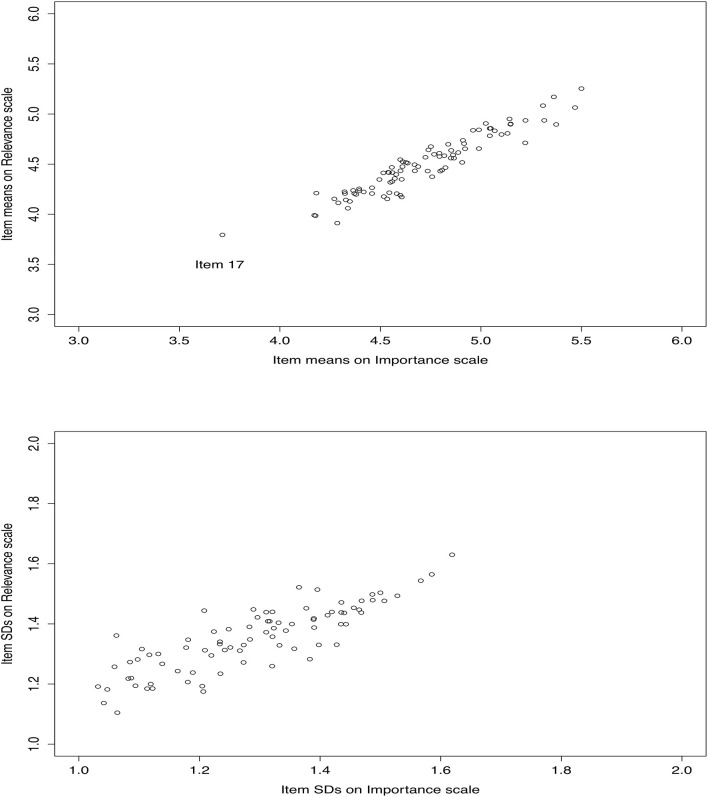
Correlation of item scores on the *How Important* section and item scores on the *How Culturally Relevant* section of the content validation rating scale for Chinese practice experts.

The authors reviewed the results of the statistical analyses and selected items based on a combination of statistical evidence and clinical considerations. First, the correlation of item scores between the *How Culturally Relevant* section and the *How Important* section (see Figure 1) indicated a relatively clear pattern other than for one item, which was standing out and was subsequently deleted as an outlier. In general, all items were considered, on average, high on both the *How Culturally Relevant* section and the *How Important* section; and the higher rating on the *How Important* section, the higher rating on the *How Culturally Relevant* section. Second, 13 items were removed or further developed because one of the following statistical criteria was met: (a) items among the lowest-ranking items, (b) items with corrected item-total correlations below 0.20, and (c) items with an item-to-component coefficient smaller than/0.60/on any rotated component. Third, a judgmental approach geared toward minimizing item redundancy and creating a briefer instrument was used. The retained items were reviewed for redundancy and were removed or reworded when appropriate, and similar or repetitive items were combined into one item. These item reduction techniques reduced the SETP-C from 89 to 67 items. The retained or reworded 67 items formed the revised version of the SETP-C. These items were categorized under five dimensions: (a) Building nurturing and responsive relationships (10 items), (b) Creating a high-quality supportive classroom environment (11 items), (c) Explicit instruction on social or emotional skills (31 items), (d) Addressing challenging behavior (9 items), and (e) Supporting family use of social-emotional practices (6 items).

### Phase 3: Content Validation With Chinese Research Experts

In this phase, conceptual and content validation with a panel of Chinese research experts who held early childhood faculty positions was conducted.

#### Participants and Interview

Five Chinese research experts from five Chinese universities who have expertise in social education, preschool curriculum, or theory of measurement in early childhood and who had not been involved in the construction of the SETP-C were invited to participate in an interview. Four of them had earned doctoral degrees in early childhood education and one had received a doctoral degree in research and evaluation methodology.

Interviews were administered individually with each research expert either during a phone or face-to-face meeting. During the interviews, the first author introduced the SETP-C to these research experts, including detailed information about its development and conceptual framework, definitions of the underlying construct and its hypothesized dimensions. Then, research experts were asked to answer a series of questions associated with two distinct areas: (a) conceptual basis of the SETP-C (including questions related to cultural relevance, under-representation, over-representation, and unfairness); and (b) soundness of the proposed use and interpretation of the SETP-C scores. After interviews, these research experts were asked to evaluate each item by giving the item a rating of 1 (*clearly measuring*), -1 (*clearly not measuring*), or 0 (*unclear*) for each dimension, and the index of item-objective congruence (Rovinelli and Hambleton, 1977) was calculated based on these experts’ ratings. Furthermore, these research experts were asked to provide feedback about the set of 67 items (including wording and clarity) and identify additional items related to the construct of interest using an open-ended question format.

#### Analyses and Results

Information obtained from interviews was analyzed qualitatively by summarizing the most common comments and highlighting meaningful suggested revisions to be made. All five Chinese research experts considered the SETP-C as a culturally appropriate instrument to measure preschool social-emotional practices, and expressed that they were impressed with the comprehensive processes used to develop the SETP-C. They strongly supported the conceptualization of social-emotional practices reflected on the SETP-C, which was based on the *Pyramid Model* and two Chinese early childhood learning standards documents for the target population (i.e., Chinese preschool teachers). Suggested edits and recommended revisions were also made by these research experts for the further refinement of the SETP-C. These recommended revisions included (a) improving the wording of some SETP-C items and their translation into Chinese (e.g., items were too long, double-barred items, ambiguous wording); (b) dividing the dimension of explicit instruction on social or emotional skills into two dimensions, social-emotional instructional content (e.g., “I explicitly teach children friendship skills) and social-emotional instructional strategies (e.g., “I use role-playing to teach children positive social or emotional skills”); and (c) distinguishing teaching practices related to using effective strategies to respond to low-intensity challenging behavior from those focused on practices for children with persistent challenging behavior.

Changes to the revised version of the SETP-C were then made based on the feedback received from these Chinese research experts. In addition, after considering issues and concerns raised about the quality of translation, the priority in the next phase of cognitive interviewing shifted to the linguistic interpretation and translation of the SETP-C.

### Phase 4: Response Process Validation Using Cognitive Interviews

The SETP-C was initially written in English (source language) and then translated into Chinese (target language), it is important to establish functional equivalence of items between the source and target languages, as well as the adequacy and appropriateness of the translation for the target culture (Cheung and Cheung, 2003; van de Vijver and Tanzer, 2004).

#### Participants and Measure

A total of 10 Chinese in-service and preservice preschool teachers were involved in cognitive interviewing. The aim of cognitive interview was to gather information about the cognitive processes that respondents used to answer the questionnaire items, which provided in-depth insight into possible misinterpretation of the translated items and cultural differences in the interpretation.

Sixty-seven items in the revised version of the SETP-C were the focus of the cognitive interview, and special attention was given to 35 items because they were identified as confusing items by the Chinese research experts, were double-barreled items, or seemed more difficult for respondents to answer than the other items. A combination of think-aloud and verbal probing methods were used. Different types of verbal probes adapted from Willis (2005) were used, including meaning-oriented probe, paraphrasing, process-oriented probe, evaluative probe, elaborative probe, hypothetical probe, recall probe, translation-oriented probe, and fairness-oriented probe. Immediately after the cognitive interviewing, these preschool teachers were asked to provide written comments on the SETP-C.

#### Analyses and Results

Based on information gathered through the cognitive interviewing and written comments, the first author modified the revised version of the SETP-C (both English and Chinese version), which was then subjected to scrutiny by the second and fifth authors. Several iterations of revisions were conducted and then reviewed, with ongoing modifications and edits being made to the questionnaire after each round. Almost all items were retranslated to promote the appropriate and accurate interpretation by respondents. Particular attention was placed on double-barreled items, items/phrases that were reported to be confusing to respondents during the cognitive interviewing, frequently used phrases throughout the instrument, and culture-specific connotations of phrases. When not violating practices associated with the *Pyramid Model* and China’s ELDG and GPE documents, double-barreled items were modified to reflect one practice. For example, the item “I join in children’s play and engage in conversations about their play” was modified to read “While they are playing, I talk with children about their play.” Another item that respondents identified as confusing, “I use peer-mediated strategies to support peers to learn and practice pro-social behaviors for use with their classmates who have social skills delays,” was reworded and separated into two items. The two revised items were “I teach peers strategies about how to interact with their classmates with social skills delays,” and “I support peers to use pro-social behaviors with their classmates who have social skills delays.” The phrase “explicitly teach” was used in more than 10 items, which was translated with slight variation across items, such as 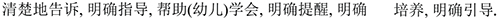 However, “teach” was not literally translated as 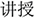 or 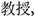 because 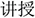 and 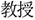 have a strict connotation in the Chinese preschool contexts that emphasizes a traditional academic approach and learning is narrowed to only academics (Zhu, 2007). Followed by interactive discussions and ongoing modifications, a 70-item version of the SETP-C was finalized for further psychometric analyses in Study 2.

## Study 2: Preliminary Psychometric Analyses of the SETP-C Scores

The validation activities and evidence described above resulted in the final version of the SETP-C used in study 2. Regarding the internal structure of the SETP-C, a five-dimension construct was originally hypothesized. Two of these dimensions, however, were suggested to be further divided into two separate dimensions during external expert. Accordingly, we specified four potential measurement models that were based on all possible combinations of the initial five dimensions and the suggested two dimensions [i.e., five-factor, six-factor (A), six-factor (B), and seven-factor]. Dimensions were allowed to correlate in all measurement models based on the conceptual framework.

### Participants and Measure

A cross-sectional survey research design was used to gather data from Chinese preschool teachers using the final version of the SETP-C. The participants were 1,599 Chinese preschool teachers from 120 randomly sampled preschools in Beijing and Ningbo. The 120 participating preschools were selected to represent two stratification factors: economic development level of the area where the preschool was located and quality level of the preschool based on their provincial government’s quality rating system. Supplementary Table 4 presents demographic characteristics of these teachers.

The SETP-C used for this part of the study included 70 items that each describes a social-emotional teaching practice in preschool settings. On a 6-point Likert scale, the participants were asked to report their frequency of implementing (*How Often* section of the SETP-C) and the level of confidence with implementing (*How Confident* section of the SETP-C) the teaching practice covered in an item. Ratings of an item range from 1 (*almost never use/not at all confident*) to 6 (*almost always use/extremely confident*). A total score can be obtained by summing the ratings on individual items, and a higher score indicates more frequent use of or a higher level of confidence in implementing social-emotional practices.

### Analytic Procedures

To examine measurement structure underlying the SETP-C, confirmatory factor analyses (CFAs) were conducted separately for the *How Often* section and the *How Confident* section, according to the four models described earlier. Given the SETP-C items were ordered categorical variables, the weighted least squares with adjusted means and variances (WLSMV) estimator was used. In addition to the model chi-square test, the root mean square error of approximation (RMSEA), comparative fit index (CFI), and Tucker-Lewis index (TLI) were used to assess model fit. Further, as the hypothesized measurement models were nested, we compared the models based on the new scaled difference test (Satorra and Bentler, 2010) to determine the best fitting model. Significant results of the chi-square test indicate that the less restricted model fits the data better than the more restricted model. Similar to the model chi-square tests, the chi-square difference tests for nested models also depend on sample size (Brannick, 1995). Therefore, model comparisons were then performed based on a practical improvement in model-fit approach, that is, a difference of 0.01 or greater between TLI estimates as recommended by Vandenberg and Lance (2000), in combination with the chi-square difference tests. M*plus* 7.4 (Muthén and Muthén, 1998-2017) was used to fit the CFA models.

Once the best fitting CFA model with a solid theoretical and empirical basis was chosen, we calculated Cronbach’s alpha and the omega coefficient to estimate internal consistency reliability for each dimension. After confirming the unidimensionality assumption for each dimension (e.g., Ye et al., 2018), we examined item characteristics by using the graded response model (GRM; Samejima, 1997). The GRM returns estimates of a discrimination parameter (denoted as “a”) and a set of threshold parameters (denoted as “b”) for each item that enable identifications of any items that may not be performing adequately. In our study, as each item of the SETP-C incorporates six categories, there were five threshold parameters. Item fit was assessed based on the *S*−χ^2^ index (*p* < 0.05 indicates adequate fit; Orlando and Thissen, 2003) and item-level RMSEA (close to zero indicates adequate fit; Kline, 2016). The latter was used because the RMSEA quantifies the magnitude of “badness-of-fit,” and enables us to determine how much an item deviates from adequate fit when the *S*−χ^2^ suggests poor item fit. In addition, we also reviewed the item characteristic curves, item information, and test information function curves. Based on the estimates of GRM, we further examined statistical bias of items across regions by conducting differential item functioning (DIF) analysis based on the ordinal logistic regression approach (Crane et al., 2006), and *p*-value was adjusted using Bonferrroni correction (0.05/item number of a particular dimension). The region variable consists of two categories, urban and rural, that designate the location of sampled preschools. We defined the urban subgroup as the reference and the rural subgroup as the focal group in the DIF analysis. All the analyses were completed in R 4.0.3 (R Core Team, 2020) where we used the mirt package (Chalmers, 2012) for item characteristics and the lordif (Choi et al., 2011) for DIF analysis.

### Results

Tables 1, 2 summarize the model fit indices for the four CFA models on the *How Often* section and *How Confident* section, respectively. The RMSEA, CFI, and TLI estimates for the seven-factor model met the recommended criteria (Hu and Bentler, 1999), indicating adequate fit of the model to the data on both the *How Often* (RMSEA = 0.044, CFI = 0.966, TLI = 0.965) and *How Confident* sections (RMSEA = 0.042, CFI = 0.968, TLI = 0.967). When compared with the other three models, the seven-factor model had the lowest RMSEA value, and the highest CFI and TLI values.

**TABLE 1:** Fit indices for the proposed four models on the *How Often* section.

**Model**	**Specifications**	**χ^2^**	***df***	***p*-Value**	**RMSEA [CI]**	**CFI**	**TLI**
Model 1: Five-factor	1. Nurturing and responsive relationships 2. Supportive classroom environment 3. Social-emotional instruction 4. Addressing CB 5. Supporting family	18270.942	2335	<0.001	0.065 [0.064–0.066]	0.925	0.922
Model 2: Six-factor (A)	1. Nurturing and responsive relationships 2. Supportive classroom environment 3. Social-emotional instruction 4. Responses to CB 5. Interventions for children with persistent CB 6. Supporting family	12639.009	2330	<0.001	0.053 [0.052–0.054]	0.951	0.949
Model 3: Six-factor (B)	1. Nurturing and responsive relationships 2. Supportive classroom environment 3. Social-emotional instructional content 4. Social-emotional instructional strategies 5. Addressing CB 6. Supporting family	15758.962	2330	<0.001	0.060 [0.059–0.061]	0.936	0.934
Model 4: Seven-factor	1. Nurturing and responsive relationships 2. Supportive classroom environment 3. Social-emotional instructional content 4. Social-emotional instructional strategies 5. Responses to CB 6. Interventions for children with persistent CB 7. Supporting family	9442.411	2324	<0.001	0.044 [0.043–0.045]	0.966	0.965

*CB, challenging behavior; *df*, degree of freedom; RMSEA, root mean square error of approximation; CI, confidence interval; CFI, comparative fit index; TLI, Tucker-Lewis index.*

**TABLE 2:** Fit indices for the proposed four models on the *How Confident* section.

**Model**	**Specifications**	**χ^2^**	***df***	***p*-Value**	**RMSEA [CI]**	**CFI**	**TLI**
Model 1: Five-factor	1. Nurturing and responsive relationships 2. Supportive classroom environment 3. Social-emotional instruction 4. Addressing CB 5. Supporting family	17196.641	2335	<0.001	0.063 [0.062–0.064]	0.929	0.926
Model 2: Six-factor (A)	1. Nurturing and responsive relationships 2. Supportive classroom environment 3. Social-emotional instruction 4. Responses to CB 5. Interventions for children with persistent CB 6. Supporting family	11536.643	2330	<0.001	0.050 [0.049–0.051]	0.956	0.954
Model 3: Six-factor (B)	1. Nurturing and responsive relationships 2. Supportive classroom environment 3. Social-emotional instructional content 4. Social-emotional instructional strategies 5. Addressing CB 6. Supporting family	15131.86	2330	<0.001	0.059 [0.058–0.060]	0.939	0.936
Model 4: Seven-factor	1. Nurturing and responsive relationships 2. Supportive classroom environment 3. Social-emotional instructional content 4. Social-emotional instructional strategies 5. Responses to CB 6. Interventions for children with persistent CB 7. Supporting family	8912.503	2324	<0.001	0.042 [0.041–0.043]	0.968	0.967

*CB, challenging behavior; *df*, degree of freedom; RMSEA, root mean square error of approximation; CI, confidence interval; CFI, comparative fit index; TLI, Tucker-Lewis index.*

Given this evidence, it was determined that the seven-factor model served as the baseline model against which all other three models were compared using chi-square difference model comparison tests. The comparisons of the seven-factor model with the other three models revealed that the seven-factor model explained the data better than the others in this sample of Chinese preschool teachers, on both the *How Often* and *How Confident* sections. Specifically, on the *How Often* section, there was a statistically significant improved fit for the seven-factor model when compared with the five-factor model, Δχ^2^ (11) = 2496.849, *p* < 0.001; first six-factor model (A), Δχ^2^ (6) = 1036.162, *p* < 0.001; and second six-factor model (B), Δχ^2^ (6) = 1676.009, *p* < 0.001. The seven-factor model was found to be practically a better fit than the other three models, with a ΔTLI of 0.043, 0.016, and 0.031, respectively. With respect to the *How Confident* section, the seven-factor model demonstrated a statistically and practically significant improvement in model fit as compared to the five-factor model, Δχ^2^ (11) = 2645.780, *p* < 0.001, ΔTLI = 0.041; first six-factor model (A), Δχ^2^ (6) = 958.617, *p* < 0.001, ΔTLI = 0.013; and second six-factor model (B), Δχ^2^ (6) = 1969.970, *p* < 0.001, ΔTLI = 0.031.

From these results, it appeared the seven-factor model best represented the internal structure of the SETP-C in the present study and was ultimately chosen because of its conceptual and statistical soundness. With respect to the standardized factor loadings for each latent variable based on the seven-factor model, all were statistically significant, ranged from 0.586 to 0.950 on the *How Often* section and 0.643 to 0.962 on the *How Confident* section, as shown in Supplementary Table 5.

We calculated the Cronbach’s alpha and omega coefficients for the seven dimensions. On the *How Often* section, Cronbach’s alpha was in the excellent range (α ≥ 0.9) for almost all dimensions except one in the good range (0.9 > α ≥ 0.8), indicating high internal consistency. For each dimension, omega coefficient (all exceeded 0.90) was slightly higher than the Cronbach’s alpha coefficient, providing further evidence for high score reliability. A similar pattern was observed for these seven dimensions on the *How Confident* section, with alpha coefficients ranging from 0.891 to 0.967 and omega coefficients ranged from 0.921 to 0.977.

**FIGURE 2:**
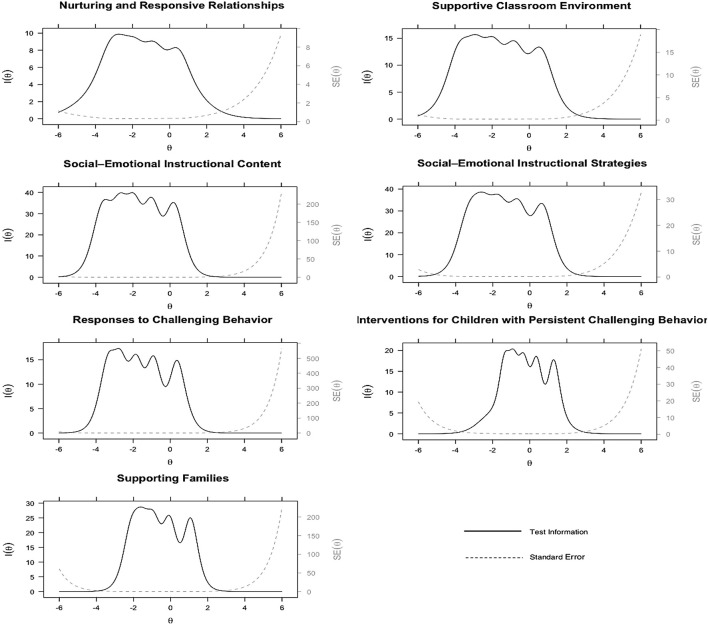
Test information function curves for the seven SETP-C dimensions on the *How Often* section.

**FIGURE 3:**
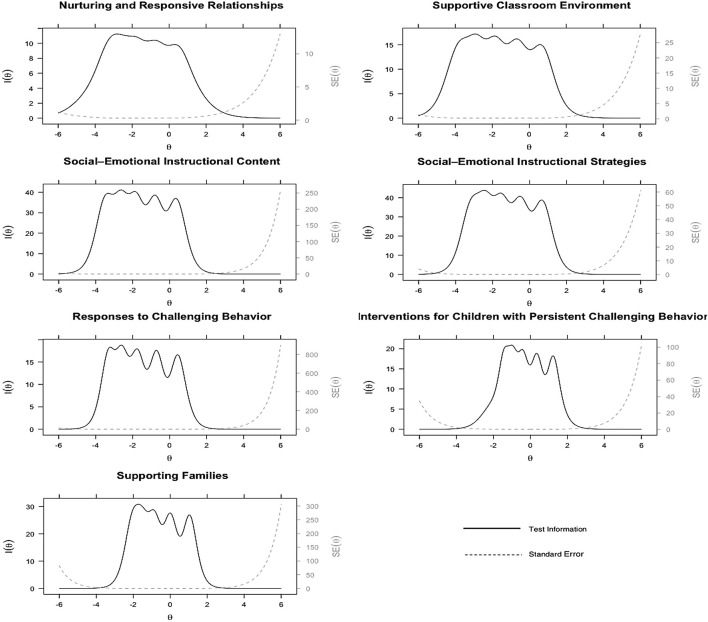
Test information function curves for the seven SETP-C dimensions on the *How Confident* section.

Results of GSM analyses are presented in the second (IRT item discrimination and difficulty) and third (IRT item fit) panels of Supplementary Table 5 and Figures 2, 3. According to the item fit information, there were items displayed statistical significance based on the *S*−χ^2^ test, indicating poor fit. However, the RMSEA values of these items were close to zero, which suggests only a minimal amount of deviance from adequate fit. Collectively, we concluded the items fit adequately.

As shown in the second panel of Supplementary Table 5, estimates of the slope parameters ranged from 1.25 to 5.55 on the *How Often* section (and ranged from 1.53 to 5.52 on the *How Confident* section), indicating a strong association between the item and the latent variable they were designed to measure. In addition, estimates of the category threshold parameters for the SETP-C ranged from −4.83 to 1.53 on the *How Often* section (and ranged from −4.91 to 1.68 on the *How Confident* section).

According to the test information function curves for the seven SETP-C dimensions on the *How Often* (Figure 2) and *How Confident* (Figure 3) sections, each dimension displayed a wide coverage of the ability on the corresponding dimension, approximately between -3 and 1 for five dimensions (i.e., Nurturing and Responsive Relationships, Supportive Classroom Environment, Social-Emotional Instructional Content, Social-Emotional Instructional Strategies, and Responses to Challenging Behavior) and between -2 and 1 for the other two dimensions (i.e., Interventions for Children with Persistent Challenging Behavior, and Supporting Family). Further, when ability level exceeded the mentioned ranges, the information function began to decline gradually (standard error of measurement increases accordingly). This implies that the SETP-C dimensions provide a precise measure for teachers whose ability levels are from below to moderately above the average.

With respect to the DIF results, on the *How Often* section, items 1 and 3 (Nurturing and Responsive Relationships dimension) and item 66 (Supporting Family dimension) were flagged for DIF. For the *How Confident* section, items 8 and 9 of the Nurturing and Responsive Relationships dimension and item 54 of the Responses to Challenging Behavior dimension were marked for DIF. This suggested that regions were significantly associated with preschool teachers’ responses on these items.

## Discussion

The SETP-C was designed as a self-report instrument to measure Chinese preschool teachers’ perspectives about the frequency of use and confidence with implementing social-emotional practices. Following DeVellis (2012) instrument development guidelines, four phases of investigation were used to develop and validate the use of the SETP-C. Various sources of validity evidence were gathered using systematic and iterative quantitative and qualitative approaches, particularly through the cultural and practice lenses of Chinese early childhood professionals. The results yielded consistent evidence in favor of a seven-factor model interpretation of the SETP-C, suggesting the SETP-C provides a measure of seven latent variables related to preschool social-emotional practices. Seven correlated latent variables/dimensions were identified and labeled: (a) Nurturing and Responsive Relationships; (b) Supportive Classroom Environment; (c) Social-Emotional Instructional Content; (d) Social-Emotional Instructional Strategies; (e) Responses to Challenging Behavior; (f) Interventions for Children with Persistent Challenging Behavior; and (g) Supporting Family Use of Social-Emotional Practices.

Given the SETP-C was developed and administered for the first time in the present study, it is not possible to directly compare and contrast findings regarding psychometric evidence reported in the present study and evidence from previous studies. Findings are interpreted with respect to the very limited number of existing studies conducted using similar self-report measurement instruments. Although the item sets for the present study differed from those used in other studies, a similar and comparable construct was found as did in other studies. For example, Heo et al. (2014) developed a questionnaire based on 38 items included on the TPOT to assess Korean early childhood teachers’ perspectives about the importance and implementation of social-emotional practices associated with the *Pyramid Model*. Exploratory factor analysis was conducted using data from a sample of 256 Korean early childhood teachers, and four latent variables were identified and labeled (a) Responsive Interactions, (b) Classroom Preventive Practices, (c) Social-Emotional Teaching Strategies, and (d) Individualized Interventions (Heo et al., 2014). Close inspection of questionnaire items associated with these four latent variables suggest they were generally comparable to the Nurturing and Responsive Relationships, Supportive Classroom Environment, Social-Emotional Instructional Content, and Interventions for Children with Persistent Challenging Behavior dimensions that were found in the present study.

An examination of the factor loadings for each item associated with one of the latent variables proposed in the seven-factor model indicated strong and statistically significant associations between items and latent variables, suggesting these items are good indicators of the latent variables intended to be measured. Given substantial efforts have been undertaken in the development and validation of the SETP-C, it was not unexpected that items on the SETP-C appear to be good indicators of the latent variables. Furthermore, the data presented in this study provide evidence for the reliability of the SETP-C scores by demonstrating acceptable internal consistency reliability for each dimension.

The results from GRM analyses further demonstrated the technical adequacy of SETP-C items. Specifically, items on all seven dimensions presented adequate item-level fit to the response data, and estimates of the slope parameter and category threshold parameters were within acceptable range. Additionally, the test information function also suggests superior accuracy in measuring social-emotional instruction of Chinese preschool teachers with latent trait levels between -3 and 1. The SETP-C items adequately capture social-emotional instruction of Chinese preschool teachers with moderate and low levels of competencies. In the present study, DIF was found on three out of 70 items within each section (i.e., *How Often*, *How Confident*) of the SETP-C. According to Holland and Wainer (2012), 10-20% of the DIF items are reasonable and acceptable.

Taken together, findings from these preliminary psychometric analyses of the SETP-C scores suggest the SETP-C provides an adequate measure of preschool social-emotional practices, and our findings also provide evidence of acceptable psychometric properties of the SETP-C.

## Limitations and Implications

Several limitations and delimitations of the present study are noted. First, development and validation of the SETP-C was primarily focused on gathering content and structural validity evidence in the Chinese socio-cultural context. Validity evidence based on SETP-C relations to other variables was not addressed given the scope of the current study. Second, although a nationwide geographically representative sample was involved in the content validation of the SETP-C (i.e., practice experts in phase 2), teachers recruited for the present study were preschool teachers from two economically powerful metropolises in mainland China. Further validation of the SETP-C that involves early childhood teacher participants from more diverse economic, geographic, program, and educational backgrounds in China will provide important incremental validity evidence to enhance applicability and generalizability of data obtained. Third, the present study does not provide information about the consistency of the SETP-C scores over time, given this study only involved cross-sectional data collection. Fourth, a short form of the SETP-C could be investigated to reduce response burden, given the current version of the SETP-C contained 70 teaching practice items being rated twice for frequency and confidence. Fifth, we used GRM for item analysis. However, given the nature of dimensionality of the SETP-C, this unidimensional IRT model may not be able to provide a comprehensive understanding of the psychometric properties of the items. Hence, given the advantages of using multidimensional item response theory (MIRT) models (e.g., Reckase, 2009; Ye et al., 2019), we encourage researchers using MIRT models in future validation studies of the SETP-C.

The findings have several potential implications for practice and research. As a self-report measure, the SETP-C is easy to administer, and relies on teachers’ subjective evaluation of and reflect on their implementation of social-emotional practices. These practices included on the SETP-C could be used to inform and organize Chinese preschool teachers’ classroom practices for working with young children and their families. The SETP-C might provide Chinese teachers with a framework for understanding the domains of their teaching practices aimed at promoting the SEC of young children. Further, the SETP-C might be used in conjunction with direct observations of practice to provide Chinese teachers with opportunities to reflect on and receive feedback about their areas of strength and needs in implementing preschool social-emotional practices. The SETP-C could be used as a professional development tool to identity the teaching practices that are in place and areas of focus for training, coaching, or other practice implementation supports. A comprehensive set of professional development supports could be developed around each of the seven dimensions identified in the present study, and, when appropriate attention could focus on particular dimensions Chinese teachers are less likely to endorse, either for frequency or confidence. For research, the SETP-C could be used as a complement to observed practice implementation in studies designed to examine the effects of professional development on preschool teachers’ implementation of social-emotional practices and corollary relationships to children’s SEC.

## Conclusion

The SETP-C is designed to measure a comprehensive set of social-emotional practices that are shown to promote young children’s SEC and are culturally relevant in Chinese preschools. The findings of this study provide preliminary evidence for the reliability and validity of scores obtained by this practice-focused measure. In this sample of Chinese preschool teachers, the SETP-C measured seven correlated latent variables of the underlying construct that were psychometrically and conceptually distinct. To sum, the SETP-C shows promise for measuring Chinese preschool teacher’s self-reported frequency of and confidence in use of social-emotional practices.

## Data Availability Statement

The raw data supporting the conclusions of this article will be made available by the authors, without undue reservation.

## Ethics Statement

The studies involving human participants were reviewed and approved by the University of Florida Institutional Review Boards. Written informed consent for participation was not required for this study in accordance with the national legislation and the institutional requirements.

## Author Contributions

LL completed the Ph.D. thesis of the article under the supervision of the PS, AH-M, and XH. LL, PS, and XH did the conceptualization and design of the work. YQ and AH-M contributed to the data analyses and interpretation. All authors were involved in the manuscript revision.

## Conflict of Interest

The authors declare that the research was conducted in the absence of any commercial or financial relationships that could be construed as a potential conflict of interest.

## Publisher’s Note

All claims expressed in this article are solely those of the authors and do not necessarily represent those of their affiliated organizations, or those of the publisher, the editors and the reviewers. Any product that may be evaluated in this article, or claim that may be made by its manufacturer, is not guaranteed or endorsed by the publisher.
