# Supplementary material for: Development and Validation of a Questionnaire to Measure Chinese Preschool Teachers’ Implementation of Social-Emotional Practices
Source: Front Psychol. 2021 Sep 10;12:699334. doi: 10.3389/fpsyg.2021.699334 (PMC8460858; doi:10.3389/fpsyg.2021.699334)
Supplement: Supplementary file 1 [file Data_Sheet_1.docx]

Development and Validation of a Questionnaire to Measure Chinese Preschool Teachers' Implementation of Social-Emotional Practices

Supplementary Material

**Supplementary Table 1.** Characteristics of Chinese Practice Experts Involved in the Content Validation Study *(n* = 205^*^)

| Variable | | Number | Percentage (%) |
| --- | --- | --- | --- |
| Professional role | Principal | 148 | 73.6 |
|  | Vice principal | 39 | 19.4 |
|  | Teacher | 14 | 7.0 |
| Level of education | Normal school graduate ^a^ | 2 | 1.0 |
|  | Associate’s degree | 31 | 15.3 |
|  | Bachelor’s degree | 165 | 81.7 |
|  | Master’s degree | 4 | 2.0 |
| Major | Early childhood education | 119 | 58.9 |
|  | Early childhood special education | 1 | .5 |
|  | Elementary education | 21 | 10.4 |
|  | Education management and leadership | 14 | 6.9 |
|  | More than two majors | 30 | 14.9 |
|  | Other | 17 | 8.4 |
| Funding source for preschool | Public | 176 | 86.7 |
|  | Private | 19 | 9.4 |
| Use of social-emotional curricula | No | 174 | 88.3 |
|  | Yes | 23 | 11.7 |
|  |  |  |  |
|  |  | *M* | *SD* |
| Years of teaching experience | N/A (continuous) | 17.1 | 9.51 |

*Note:* ^a^ Normal school graduate is equivalent to high school graduate.

*A total of 213 Chinese practice experts returned the content validating rating scale. However, data from eight practice experts were not included in the analysis due to incomplete responses. Therefore, the final sample size was 205.

**Supplementary Table 2.** Descriptive Statistics and Score Distributions on the *How Important Section* across 89 Items Included in the Content Validation Rating Scale for Chinese Practice Experts

| Item | *n* | *M* | *SD* | Variance | Dis. | Response Category Proportions (%) | | | | | | Missing Data Rate |
| --- | --- | --- | --- | --- | --- | --- | --- | --- | --- | --- | --- | --- |
|  |  |  |  |  |  | 1 | 2 | 3 | 4 | 5 | 6 |  |
| 1 | 204 | 5.13 | 1.21 | 1.46 | .28 | 2.5 | 2.0 | 6.4 | 11.3 | 25.0 | 52.9 | .5% |
| 2 | 205 | 5.10 | 1.08 | 1.17 | .38 | .5 | 2.0 | 8.3 | 12.2 | 30.2 | 46.8 | .0% |
| 3 | 203 | 4.92 | 1.24 | 1.54 | .43 | 2.0 | 3.4 | 8.4 | 15.8 | 28.1 | 42.4 | 1.0% |
| 4 | 205 | 5.47 | .83 | .68 | .41 | .0 | .0 | 2.9 | 12.7 | 19.0 | 65.4 | .0% |
| 5 | 204 | 5.50 | .90 | .81 | .42 | .0 | 2.0 | 2.9 | 7.4 | 18.6 | 69.1 | .5% |
| 6 | 204 | 5.31 | .92 | .85 | .49 | 0.5 | .5 | 2.9 | 14.7 | 26.5 | 54.9 | .5% |
| 7 | 204 | 5.04 | 1.05 | 1.10 | .57 | .0 | 1.5 | 7.4 | 21.6 | 24.5 | 45.1 | .5% |
| 8 | 205 | 4.61 | 1.27 | 1.62 | .38 | 2.0 | 3.9 | 13.7 | 23.9 | 24.9 | 31.7 | .0% |
| 9 | 205 | 4.52 | 1.29 | 1.66 | .49 | 2.0 | 5.4 | 13.7 | 25.9 | 24.4 | 28.8 | .0% |
| 10 | 201 | 4.91 | 1.23 | 1.52 | .58 | 3.0 | 1.5 | 6.5 | 22.4 | 23.9 | 42.8 | 2.0% |
| 11 | 204 | 5.36 | .93 | .86 | .53 | .5 | 1.0 | 2.5 | 13.2 | 23.5 | 59.3 | .5% |
| 12 | 203 | 5.22 | 1.03 | 1.06 | .54 | 1.0 | 1.5 | 3.0 | 16.7 | 24.6 | 53.2 | 1.0% |
| 13 | 204 | 4.34 | 1.47 | 2.16 | .53 | 3.9 | 7.8 | 20.1 | 15.7 | 23.5 | 28.9 | .5% |
| 14 | 203 | 4.82 | 1.25 | 1.57 | .62 | 2.5 | 1.0 | 12.8 | 19.7 | 24.1 | 39.9 | 1.0% |
| 15 | 203 | 5.15 | 1.18 | 1.39 | .59 | 2.5 | 2.0 | 3.9 | 14.8 | 23.6 | 53.2 | 1.0% |
| 16 | 203 | 5.37 | .88 | .77 | .58 | .0 | .5 | 5.4 | 7.4 | 29.6 | 57.1 | 1.0% |
| 17 | 203 | 3.71 | 1.62 | 2.62 | .54 | 15.3 | 4.4 | 26.6 | 18.2 | 18.2 | 17.2 | 1.0% |
| 18 | 203 | 4.29 | 1.30 | 1.68 | .34 | 1.5 | 5.9 | 24.1 | 22.2 | 23.6 | 22.7 | 1.0% |
| 19 | 203 | 4.38 | 1.39 | 1.93 | .50 | 6.4 | 2.0 | 14.3 | 27.6 | 24.1 | 25.6 | 1.0% |
| 20 | 205 | 4.75 | 1.33 | 1.78 | .59 | 2.9 | 5.4 | 9.3 | 14.6 | 31.7 | 36.1 | .0% |
| 21 | 202 | 4.32 | 1.44 | 2.06 | .63 | 3.5 | 8.9 | 17.3 | 19.3 | 24.3 | 26.7 | 1.5% |
| 22 | 199 | 4.79 | 1.22 | 1.49 | .59 | 2.0 | 3.0 | 9.0 | 21.1 | 29.1 | 35.7 | 2.9% |
| 23 | 204 | 5.15 | 1.11 | 1.24 | .52 | 1.5 | 2.0 | 5.9 | 11.3 | 29.9 | 49.5 | .5% |
| 24 | 203 | 5.32 | .93 | .86 | .59 | .5 | 1.5 | 3.0 | 9.4 | 32.5 | 53.2 | 1.0% |
| 25 | 204 | 5.14 | 1.09 | 1.20 | .56 | 1.0 | 2.0 | 6.4 | 12.7 | 28.4 | 49.5 | .5% |
| 26 | 204 | 5.22 | 1.06 | 1.13 | .48 | 2.0 | .0 | 4.4 | 14.7 | 25.5 | 53.4 | .5% |
| 27 | 205 | 4.46 | 1.57 | 2.46 | .59 | 6.8 | 5.9 | 14.6 | 16.6 | 19.5 | 36.6 | .0% |
| 28 | 204 | 4.18 | 1.38 | 1.91 | .55 | 5.4 | 6.4 | 16.7 | 27.5 | 24.5 | 19.6 | .5% |
| 29 | 202 | 4.51 | 1.24 | 1.53 | .64 | .5 | 5.9 | 13.4 | 31.2 | 19.8 | 29.2 | 1.5% |
| 30 | 202 | 4.56 | 1.27 | 1.62 | .67 | 3.0 | 4.0 | 11.4 | 24.8 | 29.7 | 27.2 | 1.5% |
| 31 | 202 | 4.60 | 1.32 | 1.74 | .74 | 3.5 | 3.0 | 14.4 | 19.3 | 29.2 | 30.7 | 1.5% |
| 32 | 204 | 4.63 | 1.43 | 2.04 | .72 | 3.9 | 6.9 | 8.8 | 20.1 | 23.5 | 36.8 | .5% |
| 33 | 204 | 4.54 | 1.40 | 1.95 | .67 | 4.4 | 3.9 | 14.7 | 19.1 | 26.0 | 31.9 | .5% |

**Supplementary Table 2.** (continued)

| Item | *n* | *M* | *SD* | Variance | Dis. | Response Category Proportions (%) | | | | | | Missing Data Rate |
| --- | --- | --- | --- | --- | --- | --- | --- | --- | --- | --- | --- | --- |
|  |  |  |  |  |  | 1 | 2 | 3 | 4 | 5 | 6 |  |
| 34 | 205 | 4.37 | 1.44 | 2.06 | .71 | 5.4 | 5.4 | 15.1 | 22.4 | 24.4 | 27.3 | .0% |
| 35 | 203 | 4.29 | 1.47 | 2.16 | .73 | 6.4 | 5.9 | 15.3 | 22.7 | 24.1 | 25.6 | 1.0% |
| 36 | 205 | 4.46 | 1.49 | 2.21 | .75 | 4.4 | 7.3 | 15.6 | 17.1 | 22.0 | 33.7 | .0% |
| 37 | 203 | 4.57 | 1.53 | 2.34 | .73 | 6.9 | 3.4 | 14.3 | 14.3 | 23.2 | 37.9 | 1.0% |
| 38 | 204 | 4.33 | 1.44 | 2.07 | .73 | 4.9 | 6.9 | 15.7 | 22.1 | 24.0 | 26.5 | .5% |
| 39 | 204 | 4.42 | 1.47 | 2.15 | .77 | 5.4 | 4.9 | 18.1 | 15.2 | 27.0 | 29.4 | .5% |
| 40 | 203 | 4.72 | 1.32 | 1.75 | .76 | 3.4 | 4.4 | 7.9 | 19.7 | 29.6 | 35.0 | 1.0% |
| 41 | 204 | 4.50 | 1.41 | 2.00 | .73 | 4.9 | 4.9 | 12.7 | 20.6 | 27.0 | 29.9 | .5% |
| 42 | 203 | 4.18 | 1.59 | 2.51 | .72 | 8.9 | 7.4 | 15.3 | 21.2 | 20.2 | 27.1 | 1.0% |
| 43 | 204 | 4.17 | 1.49 | 2.21 | .75 | 7.4 | 5.4 | 19.6 | 21.1 | 23.5 | 23.0 | .5% |
| 44 | 203 | 4.39 | 1.51 | 2.27 | .74 | 6.9 | 5.4 | 14.3 | 17.2 | 27.1 | 29.1 | 1.0% |
| 45 | 204 | 4.32 | 1.44 | 2.08 | .74 | 5.9 | 4.9 | 15.2 | 26.5 | 20.1 | 27.5 | .5% |
| 46 | 205 | 4.69 | 1.31 | 1.73 | .78 | 2.9 | 3.9 | 10.7 | 21.5 | 25.9 | 35.1 | .0% |
| 47 | 205 | 4.60 | 1.32 | 1.75 | .71 | 2.9 | 5.4 | 10.2 | 22.9 | 27.3 | 31.2 | .0% |
| 48 | 204 | 4.56 | 1.34 | 1.80 | .79 | 3.4 | 5.9 | 10.3 | 21.1 | 30.4 | 28.9 | .5% |
| 49 | 204 | 4.67 | 1.38 | 1.90 | .73 | 3.9 | 3.9 | 12.7 | 15.2 | 28.9 | 35.3 | .5% |
| 50 | 202 | 4.55 | 1.44 | 2.06 | .80 | 5.0 | 6.4 | 8.9 | 20.3 | 27.2 | 32.2 | 1.5% |
| 51 | 204 | 4.74 | 1.33 | 1.77 | .73 | 2.9 | 4.4 | 10.8 | 16.7 | 28.4 | 36.8 | .5% |
| 52 | 204 | 4.89 | 1.25 | 1.56 | .68 | 1.5 | 2.9 | 12.3 | 14.7 | 26.0 | 42.6 | .5% |
| 53 | 204 | 5.07 | 1.14 | 1.30 | .72 | 1.0 | 4.4 | 3.4 | 14.7 | 30.9 | 45.6 | .5% |
| 54 | 205 | 5.05 | 1.12 | 1.25 | .73 | 1.0 | 3.4 | 4.9 | 15.1 | 31.7 | 43.9 | .0% |
| 55 | 203 | 4.54 | 1.39 | 1.93 | .73 | 6.4 | 3.0 | 8.4 | 22.7 | 31.5 | 28.1 | 1.0% |
| 56 | 203 | 4.56 | 1.36 | 1.84 | .77 | 3.4 | 5.4 | 11.8 | 21.2 | 27.6 | 30.5 | 1.0% |
| 57 | 205 | 5.02 | 1.06 | 1.13 | .71 | 1.0 | 1.0 | 6.8 | 19.0 | 30.2 | 42.0 | .0% |
| 58 | 204 | 4.58 | 1.32 | 1.73 | .75 | 2.9 | 4.4 | 12.7 | 22.1 | 27.5 | 30.4 | .5% |
| 59 | 203 | 4.36 | 1.42 | 2.02 | .70 | 4.4 | 7.9 | 13.8 | 19.2 | 30.0 | 24.6 | 1.0% |
| 60 | 205 | 4.40 | 1.50 | 2.25 | .74 | 7.8 | 3.9 | 12.7 | 21.0 | 25.9 | 28.8 | .0% |
| 61 | 204 | 4.91 | 1.18 | 1.40 | .73 | 1.5 | 3.4 | 7.8 | 15.7 | 33.3 | 38.2 | .5% |
| 62 | 203 | 4.67 | 1.28 | 1.65 | .76 | 2.0 | 5.9 | 9.9 | 19.2 | 31.5 | 31.5 | 1.0% |
| 63 | 204 | 4.35 | 1.46 | 2.12 | .71 | 6.9 | 4.9 | 12.7 | 23.0 | 27.0 | 25.5 | .5% |
| 64 | 203 | 4.61 | 1.28 | 1.65 | .69 | 2.0 | 5.4 | 10.8 | 24.6 | 26.1 | 31.0 | 1.0% |
| 65 | 202 | 4.86 | 1.10 | 1.21 | .76 | 1.0 | 2.5 | 8.4 | 17.8 | 38.1 | 32.2 | 1.5% |
| 66 | 203 | 4.85 | 1.19 | 1.41 | .71 | 2.0 | 3.0 | 5.9 | 23.6 | 28.1 | 37.4 | 1.0% |
| 67 | 202 | 4.77 | 1.23 | 1.52 | .75 | 2.0 | 5.0 | 5.4 | 24.3 | 28.7 | 34.7 | 1.5% |

**Supplementary Table 2.** (continued)

| Item | *n* | *M* | *SD* | Variance | Dis. | Response Category Proportions (%) | | | | | | Missing Data Rate |
| --- | --- | --- | --- | --- | --- | --- | --- | --- | --- | --- | --- | --- |
|  |  |  |  |  |  | 1 | 2 | 3 | 4 | 5 | 6 |  |
| 68 | 203 | 5.04 | 1.09 | 1.18 | .72 | .5 | 3.0 | 5.9 | 16.3 | 31.0 | 43.3 | 1.0% |
| 69 | 203 | 4.92 | 1.13 | 1.28 | .71 | 1.0 | 3.0 | 7.9 | 16.7 | 34.0 | 37.4 | 1.0% |
| 70 | 203 | 4.74 | 1.21 | 1.45 | .69 | 1.5 | 4.9 | 7.4 | 22.2 | 32.5 | 31.5 | 1.0% |
| 71 | 203 | 4.27 | 1.39 | 1.93 | .65 | 3.9 | 7.4 | 17.2 | 23.6 | 24.6 | 23.2 | 1.0% |
| 72 | 203 | 4.84 | 1.16 | 1.36 | .65 | 1.5 | 3.0 | 7.9 | 20.7 | 32.0 | 35.0 | 1.0% |
| 73 | 201 | 4.85 | 1.18 | 1.39 | .67 | 1.5 | 2.5 | 8.5 | 22.4 | 27.4 | 37.8 | 2.0% |
| 74 | 203 | 4.64 | 1.31 | 1.72 | .61 | 2.5 | 3.9 | 14.8 | 17.7 | 28.6 | 32.5 | 1.0% |
| 75 | 203 | 4.61 | 1.27 | 1.61 | .56 | 2.5 | 5.4 | 8.4 | 24.6 | 30.5 | 28.6 | 1.0% |
| 76 | 203 | 4.79 | 1.21 | 1.46 | .73 | 1.0 | 2.5 | 14.8 | 16.3 | 29.1 | 36.5 | 1.0% |
| 77 | 204 | 4.96 | 1.12 | 1.26 | .65 | 1.0 | 2.0 | 8.8 | 16.7 | 31.4 | 40.2 | .5% |
| 78 | 202 | 4.99 | 1.04 | 1.08 | .64 | .0 | 3.0 | 6.4 | 17.3 | 35.1 | 38.1 | 1.5% |
| 79 | 203 | 4.80 | 1.21 | 1.46 | .65 | 1.5 | 3.4 | 9.4 | 21.2 | 28.6 | 36.0 | 1.0% |
| 80 | 203 | 4.58 | 1.40 | 1.95 | .68 | 3.9 | 5.9 | 8.9 | 25.1 | 21.7 | 34.5 | 1.0% |
| 81 | 204 | 4.53 | 1.31 | 1.72 | .63 | 2.9 | 4.4 | 13.2 | 24.0 | 26.5 | 28.9 | .5% |
| 82 | 202 | 4.76 | 1.22 | 1.50 | .67 | 1.0 | 4.5 | 9.9 | 23.3 | 25.2 | 36.1 | 1.5% |
| 83 | 202 | 4.54 | 1.35 | 1.83 | .59 | 1.5 | 7.9 | 13.9 | 20.3 | 24.3 | 32.2 | 1.5% |
| 84 | 203 | 4.99 | 1.12 | 1.25 | .67 | .5 | 4.4 | 4.4 | 17.7 | 32.0 | 40.9 | 1.0% |
| 85 | 202 | 4.81 | 1.06 | 1.12 | .68 | .5 | 1.0 | 11.9 | 20.8 | 35.6 | 30.2 | 1.5% |
| 86 | 201 | 4.87 | 1.09 | 1.18 | .65 | 1.0 | 2.0 | 8.0 | 20.4 | 35.8 | 32.8 | 2.0% |
| 87 | 204 | 4.82 | 1.11 | 1.22 | .65 | 1.5 | 2.0 | 7.8 | 21.1 | 36.8 | 30.9 | .5% |
| 88 | 203 | 4.61 | 1.32 | 1.75 | .68 | 2.5 | 5.9 | 11.3 | 20.2 | 29.1 | 31.0 | 1.0% |
| 89 | 202 | 4.60 | 1.37 | 1.86 | .62 | 2.5 | 5.4 | 14.9 | 18.8 | 23.8 | 34.7 | 1.5% |

*Note:* Dis. = item discrimination (i.e., corrected item-total correlation).

**Supplementary Table 3.** Descriptive Statistics and Score Distributions on the *How Culturally Relevant Section* across 89 Items Included in the Content Validation Rating Scale for Chinese Practice Experts

| Item | *n* | *M* | *SD* | Variance | Dis. | Response Category Proportions (%) | | | | | | Missing Data Rate |
| --- | --- | --- | --- | --- | --- | --- | --- | --- | --- | --- | --- | --- |
|  |  |  |  |  |  | 1 | 2 | 3 | 4 | 5 | 6 |  |
| 1 | 202 | 4.81 | 1.18 | 1.38 | .41 | 1.0 | 4.0 | 8.4 | 21.3 | 30.7 | 34.7 | 1.5% |
| 2 | 201 | 4.80 | 1.22 | 1.48 | .39 | 1.5 | 2.5 | 11.4 | 21.9 | 24.9 | 37.8 | 2.0% |
| 3 | 203 | 4.70 | 1.31 | 1.72 | .47 | 3.4 | 3.0 | 10.3 | 21.7 | 26.1 | 35.5 | 1.0% |
| 4 | 202 | 5.06 | 1.12 | 1.25 | .57 | .0 | 3.5 | 8.4 | 12.9 | 28.7 | 46.5 | 1.5% |
| 5 | 205 | 5.25 | 1.02 | 1.03 | .50 | .0 | 3.5 | 8.4 | 12.9 | 28.7 | 46.5 | .0% |
| 6 | 203 | 5.08 | 1.08 | 1.16 | .54 | 1.0 | 1.5 | 5.9 | 17.2 | 28.6 | 45.8 | 1.0% |
| 7 | 202 | 4.86 | 1.18 | 1.40 | .53 | 1.5 | .5 | 13.4 | 19.8 | 25.2 | 39.6 | 1.5% |
| 8 | 204 | 4.48 | 1.33 | 1.77 | .50 | 2.0 | 6.4 | 15.7 | 23.0 | 24.0 | 28.9 | .5% |
| 9 | 204 | 4.18 | 1.45 | 2.10 | .58 | 6.4 | 6.9 | 17.2 | 23.0 | 25.5 | 21.1 | .5% |
| 10 | 202 | 4.74 | 1.33 | 1.78 | .67 | 3.0 | 4.5 | 8.4 | 23.3 | 21.8 | 39.1 | 1.5% |
| 11 | 205 | 5.17 | 1.09 | 1.19 | .59 | .0 | 3.4 | 5.9 | 14.1 | 23.4 | 53.2 | .0% |
| 12 | 204 | 4.94 | 1.19 | 1.42 | .60 | 1.5 | 3.4 | 5.9 | 21.1 | 25.5 | 42.6 | .5% |
| 13 | 201 | 4.06 | 1.44 | 2.07 | .58 | 4.5 | 10.0 | 23.4 | 19.4 | 22.9 | 19.9 | 2.0% |
| 14 | 202 | 4.58 | 1.32 | 1.75 | .60 | 3.0 | 4.5 | 11.9 | 24.3 | 24.8 | 31.7 | 1.5% |
| 15 | 203 | 4.90 | 1.21 | 1.46 | .59 | 2.5 | 1.0 | 9.4 | 19.2 | 27.1 | 40.9 | 1.0% |
| 16 | 202 | 4.90 | 1.20 | 1.44 | .49 | 1.0 | 3.0 | 11.4 | 15.3 | 28.7 | 40.6 | 1.5% |
| 17 | 204 | 3.79 | 1.63 | 2.66 | .47 | 14.2 | 5.4 | 24.0 | 18.6 | 18.6 | 19.1 | .5% |
| 18 | 204 | 3.91 | 1.42 | 2.02 | .51 | 5.4 | 10.3 | 25.5 | 22.1 | 20.1 | 16.7 | .5% |
| 19 | 202 | 4.20 | 1.41 | 2.00 | .59 | 6.9 | 4.0 | 17.8 | 25.2 | 25.7 | 20.3 | 1.5% |
| 20 | 203 | 4.67 | 1.33 | 1.77 | .57 | 3.4 | 4.9 | 9.4 | 17.2 | 33.0 | 32.0 | 1.0% |
| 21 | 200 | 4.23 | 1.47 | 2.17 | .65 | 5.0 | 9.0 | 18.0 | 18.5 | 25.5 | 24.0 | 2.4% |
| 22 | 198 | 4.58 | 1.30 | 1.68 | .66 | 2.0 | 5.6 | 13.1 | 20.7 | 29.3 | 29.3 | 3.4% |
| 23 | 204 | 4.90 | 1.19 | 1.40 | .63 | 1.0 | 2.9 | 10.3 | 17.2 | 28.4 | 40.2 | .5% |
| 24 | 204 | 4.94 | 1.15 | 1.33 | .64 | 1.0 | 2.5 | 10.3 | 14.2 | 32.4 | 39.7 | .5% |
| 25 | 204 | 4.95 | 1.19 | 1.43 | .62 | 1.5 | 2.5 | 8.8 | 17.6 | 26.0 | 43.6 | .5% |
| 26 | 205 | 4.71 | 1.36 | 1.85 | .59 | 3.9 | 3.4 | 10.2 | 20.5 | 23.9 | 38.0 | .0% |
| 27 | 204 | 4.26 | 1.54 | 2.38 | .56 | 6.4 | 8.3 | 17.2 | 17.2 | 22.5 | 28.4 | .5% |
| 28 | 204 | 4.21 | 1.28 | 1.65 | .49 | 3.9 | 5.9 | 15.7 | 30.9 | 27.0 | 16.7 | .5% |
| 29 | 201 | 4.41 | 1.23 | 1.52 | .55 | 2.0 | 3.0 | 18.9 | 27.9 | 24.4 | 23.9 | 2.0% |
| 30 | 202 | 4.42 | 1.27 | 1.62 | .61 | 2.5 | 5.9 | 12.9 | 27.7 | 28.2 | 22.8 | 1.5% |
| 31 | 200 | 4.55 | 1.26 | 1.59 | .69 | 1.5 | 4.5 | 16.0 | 22.0 | 28.0 | 28.0 | 2.4% |
| 32 | 201 | 4.52 | 1.33 | 1.77 | .65 | 3.0 | 5.0 | 14.4 | 20.9 | 28.4 | 28.4 | 2.0% |
| 33 | 203 | 4.42 | 1.33 | 1.77 | .68 | 3.0 | 3.0 | 22.2 | 20.2 | 24.6 | 27.1 | 1.0% |

**Supplementary Table 3.** (continued)

| Item | *n* | *M* | *SD* | Variance | Dis. | Response Category Proportions (%) | | | | | | Missing Data Rate |
| --- | --- | --- | --- | --- | --- | --- | --- | --- | --- | --- | --- | --- |
|  |  |  |  |  |  | 1 | 2 | 3 | 4 | 5 | 6 |  |
| 34 | 203 | 4.21 | 1.40 | 1.96 | .68 | 5.4 | 4.9 | 19.7 | 25.6 | 22.2 | 22.2 | 1.0% |
| 35 | 202 | 4.11 | 1.48 | 2.18 | .67 | 7.4 | 5.9 | 19.3 | 24.3 | 21.3 | 21.8 | 1.5% |
| 36 | 203 | 4.21 | 1.50 | 2.24 | .77 | 5.9 | 7.4 | 20.7 | 17.2 | 23.6 | 25.1 | 1.0% |
| 37 | 202 | 4.36 | 1.49 | 2.23 | .72 | 5.9 | 5.4 | 18.3 | 16.8 | 24.3 | 29.2 | 1.5% |
| 38 | 203 | 4.14 | 1.44 | 2.06 | .69 | 5.4 | 6.9 | 22.2 | 20.2 | 24.1 | 21.2 | 1.0% |
| 39 | 202 | 4.22 | 1.45 | 2.09 | .74 | 6.4 | 5.4 | 17.8 | 22.8 | 24.8 | 22.8 | 1.5% |
| 40 | 204 | 4.57 | 1.36 | 1.84 | .70 | 3.9 | 4.9 | 10.8 | 21.6 | 28.4 | 30.4 | .5% |
| 41 | 205 | 4.35 | 1.43 | 2.04 | .64 | 5.4 | 6.8 | 12.7 | 22.9 | 27.3 | 24.9 | .0% |
| 42 | 204 | 3.99 | 1.57 | 2.45 | .70 | 9.3 | 8.3 | 20.1 | 20.6 | 20.1 | 21.6 | .5% |
| 43 | 204 | 3.99 | 1.48 | 2.19 | .74 | 8.3 | 5.4 | 24.5 | 21.1 | 22.1 | 18.6 | .5% |
| 44 | 205 | 4.25 | 1.48 | 2.18 | .71 | 6.3 | 6.3 | 17.6 | 19.5 | 25.9 | 24.4 | .0% |
| 45 | 203 | 4.21 | 1.40 | 1.96 | .74 | 4.9 | 6.9 | 17.2 | 26.1 | 23.2 | 21.7 | 1.0% |
| 46 | 204 | 4.48 | 1.41 | 1.99 | .76 | 4.4 | 5.9 | 12.3 | 22.5 | 25.0 | 29.9 | .5% |
| 47 | 203 | 4.43 | 1.39 | 1.92 | .73 | 3.9 | 6.9 | 12.8 | 20.7 | 29.6 | 26.1 | 1.0% |
| 48 | 205 | 4.33 | 1.38 | 1.90 | .81 | 3.9 | 7.8 | 14.1 | 22.4 | 29.3 | 22.4 | .0% |
| 49 | 203 | 4.43 | 1.45 | 2.11 | .73 | 4.9 | 6.9 | 13.8 | 17.2 | 28.6 | 28.6 | 1.0% |
| 50 | 201 | 4.32 | 1.44 | 2.07 | .80 | 5.5 | 6.0 | 16.9 | 18.9 | 28.4 | 24.4 | 2.0% |
| 51 | 204 | 4.43 | 1.40 | 1.97 | .76 | 3.9 | 5.9 | 16.2 | 19.6 | 26.0 | 28.4 | .5% |
| 52 | 203 | 4.62 | 1.38 | 1.91 | .68 | 3.4 | 4.4 | 13.8 | 19.2 | 23.6 | 35.5 | 1.0% |
| 53 | 203 | 4.83 | 1.27 | 1.61 | .71 | 2.0 | 6.4 | 4.4 | 18.2 | 31.5 | 37.4 | 1.0% |
| 54 | 203 | 4.86 | 1.20 | 1.44 | .74 | 1.5 | 4.4 | 4.4 | 25.1 | 25.6 | 38.9 | 1.0% |
| 55 | 202 | 4.42 | 1.39 | 1.93 | .64 | 5.9 | 4.0 | 10.9 | 26.2 | 27.7 | 25.2 | 1.5% |
| 56 | 203 | 4.47 | 1.32 | 1.74 | .71 | 3.4 | 6.4 | 9.9 | 24.6 | 31.5 | 24.1 | 1.0% |
| 57 | 202 | 4.91 | 1.11 | 1.22 | .74 | 1.0 | 2.5 | 5.9 | 23.3 | 30.2 | 37.1 | 1.5% |
| 58 | 204 | 4.40 | 1.41 | 1.98 | .80 | 3.9 | 6.9 | 15.7 | 19.6 | 27.0 | 27.0 | .5% |
| 59 | 202 | 4.24 | 1.44 | 2.07 | .74 | 4.5 | 9.9 | 14.4 | 23.3 | 24.8 | 23.3 | 1.5% |
| 60 | 203 | 4.24 | 1.50 | 2.26 | .78 | 6.4 | 8.4 | 14.3 | 22.7 | 22.7 | 25.6 | 1.0% |
| 61 | 203 | 4.52 | 1.35 | 1.82 | .76 | 3.4 | 2.5 | 18.7 | 20.7 | 23.6 | 31.0 | 1.0% |
| 62 | 204 | 4.50 | 1.35 | 1.82 | .80 | 3.4 | 4.9 | 13.7 | 23.5 | 25.5 | 28.9 | .5% |
| 63 | 203 | 4.13 | 1.45 | 2.11 | .72 | 7.4 | 5.9 | 17.7 | 24.1 | 25.1 | 19.7 | 1.0% |
| 64 | 204 | 4.35 | 1.39 | 1.93 | .74 | 2.9 | 6.4 | 19.6 | 23.5 | 19.1 | 28.4 | .5% |
| 65 | 201 | 4.59 | 1.28 | 1.64 | .79 | 1.5 | 7.0 | 10.4 | 22.4 | 29.4 | 29.4 | 2.0% |
| 66 | 204 | 4.64 | 1.24 | 1.53 | .74 | 2.0 | 2.0 | 14.7 | 25.5 | 23.5 | 32.4 | .5% |
| 67 | 199 | 4.60 | 1.34 | 1.80 | .76 | 3.0 | 5.0 | 11.6 | 22.6 | 25.1 | 32.7 | 2.9% |
| 68 | 203 | 4.78 | 1.22 | 1.49 | .79 | .0 | 5.9 | 10.3 | 20.7 | 25.6 | 37.4 | 1.0% |

**Supplementary Table 3.** (continued)

| Item | *n* | *M* | *SD* | Variance | Dis. | Response Category Proportions (%) | | | | | | Missing Data Rate |
| --- | --- | --- | --- | --- | --- | --- | --- | --- | --- | --- | --- | --- |
|  |  |  |  |  |  | 1 | 2 | 3 | 4 | 5 | 6 |  |
| 69 | 202 | 4.65 | 1.30 | 1.69 | .75 | 2.0 | 5.4 | 11.9 | 19.3 | 28.7 | 32.7 | 1.5% |
| 70 | 204 | 4.64 | 1.19 | 1.42 | .70 | .5 | 4.4 | 13.7 | 22.5 | 29.4 | 29.4 | .5% |
| 71 | 202 | 4.15 | 1.42 | 2.01 | .63 | 4.5 | 8.4 | 20.3 | 22.3 | 23.3 | 21.3 | 1.5% |
| 72 | 202 | 4.70 | 1.24 | 1.55 | .60 | 2.0 | 3.0 | 12.9 | 20.3 | 29.2 | 32.7 | 1.5% |
| 73 | 200 | 4.56 | 1.32 | 1.75 | .72 | 2.0 | 7.0 | 11.0 | 23.5 | 26.0 | 30.5 | 2.4% |
| 74 | 202 | 4.51 | 1.37 | 1.88 | .64 | 4.5 | 4.5 | 11.4 | 24.8 | 25.2 | 29.7 | 1.5% |
| 75 | 204 | 4.52 | 1.31 | 1.72 | .54 | 2.9 | 4.4 | 14.2 | 22.1 | 28.4 | 27.9 | .5% |
| 76 | 202 | 4.61 | 1.31 | 1.72 | .69 | 2.0 | 5.0 | 14.9 | 18.8 | 27.2 | 32.2 | 1.5% |
| 77 | 203 | 4.84 | 1.19 | 1.40 | .65 | 1.0 | 3.0 | 11.3 | 17.7 | 30.0 | 36.9 | 1.0% |
| 78 | 203 | 4.84 | 1.14 | 1.29 | .70 | .0 | 4.4 | 8.9 | 20.2 | 31.0 | 35.5 | 1.0% |
| 79 | 200 | 4.43 | 1.44 | 2.09 | .66 | 4.0 | 7.0 | 15.5 | 20.0 | 22.5 | 31.0 | 2.4% |
| 80 | 204 | 4.21 | 1.51 | 2.29 | .62 | 6.4 | 8.3 | 15.7 | 24.5 | 18.1 | 27.0 | .5% |
| 81 | 203 | 4.15 | 1.44 | 2.07 | .63 | 4.4 | 8.9 | 19.7 | 24.6 | 18.7 | 23.6 | 1.0% |
| 82 | 203 | 4.37 | 1.37 | 1.89 | .67 | 2.5 | 8.4 | 15.8 | 22.7 | 24.1 | 26.6 | 1.0% |
| 83 | 200 | 4.22 | 1.40 | 1.96 | .60 | 2.5 | 9.5 | 21.0 | 22.5 | 20.0 | 24.5 | 2.4% |
| 84 | 203 | 4.66 | 1.30 | 1.68 | .72 | 1.5 | 4.9 | 14.8 | 18.2 | 26.6 | 34.0 | 1.0% |
| 85 | 202 | 4.44 | 1.26 | 1.58 | .72 | 1.0 | 5.9 | 17.8 | 23.3 | 27.2 | 24.8 | 1.5% |
| 86 | 202 | 4.56 | 1.27 | 1.62 | .70 | 1.5 | 5.9 | 13.9 | 20.3 | 30.7 | 27.7 | 1.5% |
| 87 | 202 | 4.47 | 1.32 | 1.73 | .70 | 2.0 | 6.4 | 16.3 | 19.8 | 29.2 | 26.2 | 1.5% |
| 88 | 204 | 4.17 | 1.44 | 2.07 | .74 | 5.9 | 6.4 | 19.6 | 23.0 | 23.0 | 22.1 | .5% |
| 89 | 200 | 4.19 | 1.52 | 2.32 | .68 | 6.0 | 10.5 | 14.0 | 23.5 | 20.0 | 26.0 | 2.4% |

*Note:* Dis. = item discrimination (i.e., corrected item-total correlation).

**Supplementary Table 4.** Characteristics of Participating Chinese Preschool Teachers in Study 2 (*n* = 1,599)

| Variable | | Number | Percentage (%) |
| --- | --- | --- | --- |
| Region of preschool | Urban | 1135 | 71.0 |
|  | Rural | 464 | 29.0 |
| Funding source for preschool | Public | 1304 | 81.6 |
|  | Private | 295 | 18.4 |
| Quality rating of preschool | Excellent | 714 | 44.7 |
|  | Good | 738 | 46.2 |
|  | No Rating | 147 | 9.2 |
| Role of teacher | Lead Teacher | 813 | 50.8 |
|  | Assistant Teacher | 569 | 35.6 |
|  | Others | 217 | 13.6 |
| Professional title | Yes | 990 | 61.9 |
|  | No | 608 | 38.0 |
| Level of education | Bachelor or Above | 1008 | 63.0 |
|  | Below Associate | 52 | 3.3 |
|  | Associate | 539 | 33.7 |
| Major | ECE | 1442 | 90.2 |
|  | Not ECE | 157 | 9.8 |
| Teaching certification | Yes | 1524 | 95.3 |
|  | No | 75 | 4.7 |
| Age group of children | 3-4 years | 445 | 27.8 |
|  | 4-5 years | 470 | 29.4 |
|  | 5-6 years | 460 | 28.8 |
|  | 6-7 years | 200 | 12.5 |
|  | Mixed ages | 23 | 1.4 |
| Inclusion of children with disabilities | Yes | 256 | 16.0 |
|  | No | 1340 | 83.8 |
| Enrollment of children with persistent CB | Yes | 1013 | 63.4 |
|  | No | 574 | 35.9 |
| Use of social-emotional curriculum | Yes | 88 | 5.5 |
|  | No | 1511 | 94.5 |
|  |  |  |  |
|  |  | *M* | *SD* |
| Years of teaching experience | N/A (continuous) | 8.6 | 7.7 |
| Child-to-teacher ratio | N/A (continuous) | 10.3:1 | 3.1:1 |

*Note:* ECE = early childhood education; CB = challenging behavior.

# Supplementary Table 5. Results from the Accepted Confirmatory Factor Analysis Model and the Graded Response Model

| Factor/  Dimension | Item | Factor loadings | IRT item discrimination and difficulty | | | | | | IRT item fit | | | |
| --- | --- | --- | --- | --- | --- | --- | --- | --- | --- | --- | --- | --- |
|  |  |  | a | b1 | b2 | b3 | b4 | b5 | RMSEA | $S\text{-}\chi^{2}$ | $S\text{-}\chi^{2}$ *df* | $S\text{-}\chi^{2}$ *p* |
| Nurturing and Responsive Relationships (9 items; Items 1 to 9) | 1^*^ | .59 (.64) | 1.25 (1.53) | -4.81  (-4.91) | -3.35  (-3.41) | -2.10  (-1.85) | -.88  (-.14) | 1.49 (1.44) | .01 (.02) | 77.27 (88.32) | 73 (57) | .34 (.00) |
|  | 2 | .68 (.73) | 1.43 (1.80) | -4.83  (-4.40) | -3.40  (-3.06) | -2.33  (-1.90) | -.86  (-.36) | 1.15 (1.15) | .00 (.01) | 64.73 (70.12) | 64 (55) | .45 (.08) |
|  | 3^*^ | .68 (.71) | 1.45 (1.56) | -4.11  (-4.06) | -3.11  (-2.89) | -1.97  (-1.54) | -.41  (-.03) | 1.49 (1.68) | .01 (.00) | 75.77 (62.81) | 69 (65) | .27 (.55) |
|  | 4 | .69 (.74) | 1.72 (2.07) | -3.27  (-3.18) | -2.68  (-2.72) | -1.86  (-1.80) | -.93  (-.80) | .39 (.46) | .02 (.02) | 95.84 (99.45) | 70 (60) | .02 (.00) |
|  | 5 | .77 (.79) | 2.03 (2.44) | -3.47  (-3.44) | -2.91  (-2.87) | -2.07  (-1.89) | -1.11 (-.90) | .26 (.32) | .00 (.01) | 58.11 (56.03) | 58 (49) | .47 (.23) |
|  | 6 | .69 (.71) | 1.65 (1.88) | -3.42  (-3.59) | -2.80  (-2.84) | -2.06  (-2.00) | -1.15 (-.98) | .01 (.16) | .02 (.02) | 108.09 (91.11) | 72 (64) | .00 (.01) |
|  | 7 | .68 (.72) | 1.72 (1.86) | -3.29  (-3.35) | -2.74  (-2.82) | -1.75  (-1.84) | -.54  (-.54) | .89 (.87) | .01 (.02) | 84.37 (90.05) | 71 (58) | .13 (.00) |
|  | 8^♦^ | .85 (.85) | 2.71 (2.49) | -3.13  (-3.17) | -2.63  (-2.57) | -1.90  (-1.76) | -.84  (-.66) | .43 (.57) | .02 (.02) | 74.00 (86.23) | 47 (55) | .01 (.00) |
|  | 9^♦^ | .83 (.85) | 2.52 (2.48) | -3.06  (-3.07) | -2.72  (-2.50) | -1.90  (-1.69) | -.82  (-.67) | .53 (.62) | .01 (.01) | 51.65 (60.64) | 49 (55) | .37 (.28) |
| Supportive Classroom Environment  (11 items; Items 10 to 20) | 10 | .67 (.70) | 1.40 (1.56) | -3.65  (-3.75) | -2.75  (-2.75) | -1.61  (-1.65) | -.36  (-.35) | .92 (.92) | .01 (.02) | 108.63 (118.09) | 83 (84) | .03 (.01) |
|  | 11 | .75 (.74) | 2.00 (2.12) | -3.64  (-3.50) | -2.98  (-2.90) | -2.24  (-2.10) | -1.25  (-1.02) | .18 (.26) | .01 (.01) | 71.19 (87.07) | 67 (65) | .34 (.04) |
|  | 12 | .69 (.71) | 1.91 (2.08) | -3.52  (-3.57) | -2.66  (-2.75) | -1.83  (-1.82) | -.81  (-.69) | .51 (.57) | .02 (.00) | 136.41 (64.97) | 76 (64) | .00 (.44) |
|  | 13 | .75 (.77) | 2.12 (2.43) | -3.72  (-3.68) | -2.94  (-2.83) | -2.02  (-1.92) | -1.04 (-.91) | .27 (.31) | .01 (.00) | 86.51 (55.11) | 64 (59) | .03 (.62) |
|  | 14 | .78 (.79) | 2.29 (2.49) | -3.90  (-4.19) | -2.83  (-2.86) | -1.86  (-1.71) | -.62  (-.48) | .87 (.85) | .01 (.00) | 57.74 (55.29) | 53 (54) | .30 (.43) |
|  | 15 | .80 (.80) | 2.63 (2.52) | -3.65  (-4.00) | -2.95  (-2.79) | -2.02  (-1.79) | -.75  (-.58) | .75 (.86) | .01 (.02) | 62.54 (77.05) | 49 (54) | .09 (.02) |
|  | 16 | .84 (.86) | 2.95 (3.02) | -3.92  (-3.57) | -2.86  (-2.81) | -1.86  (-1.74) | -.69  (-.53) | .76 (.84) | .01 (.01) | 57.52 (48.81) | 44 (45) | .08 (.32) |
|  | 17 | .83 (.83) | 3.02 (2.96) | -3.56  (-3.87) | -2.84  (-2.92) | -1.97  (-1.90) | -.90  (-.77) | .41 (.51) | .02 (.01) | 77.60 (53.96) | 47 (46) | .00 (.20) |
|  | 18 | .69 (.75) | 1.67 (1.98) | -4.35  (-4.06) | -3.47  (-3.15) | -2.15  (-2.04) | -.85  (-.65) | .81 (.83) | .01 (.01) | 67.35 (78.44) | 62 (58) | .30 (.04) |

**Supplementary Table 5.** (continued)

| Factor/  Subscale | Item | Factor loadings | IRT item discrimination and difficulty | | | | | | IRT item fit | | | |
| --- | --- | --- | --- | --- | --- | --- | --- | --- | --- | --- | --- | --- |
|  |  |  | a | b1 | b2 | b3 | b4 | b5 | RMSEA | $S\text{-}\chi^{2}$ | $S\text{-}\chi^{2}$ *df* | $S\text{-}\chi^{2}$ *p* |
|  | 19 | .80 (.82) | 2.08 (2.29) | -4.01  (-4.09) | -3.13  (-3.13) | -2.11  (-1.97) | -.88  (-.71) | .64 (.71) | .02 (.01) | 79.65 (61.56) | 56 (55) | .02 (.25) |
|  | 20 | .75 (.79) | 1.70 (2.02) | -4.19  (-4.00) | -3.28  (-3.14) | -2.16  (-1.90) | -.85  (-.63) | .79 (.85) | .00 (.01) | 64.97 (74.16) | 65 (57) | .48 (.06) |
| Social-Emotional Instructional Content  (15 items; Items 21 to 35) | 21 | .80 (.82) | 2.35 (2.46) | -3.95  (-3.82) | -2.98  (-2.88) | -2.18  (-2.10) | -1.22 (-.98) | .12 (.24) | .02 (.02) | 106.01 (128.81) | 66 (66) | .00 (.00) |
|  | 22 | .86 (.84) | 2.75 (2.69) | -3.65  (-3.69) | -2.95  (-3.02) | -2.11  (-2.04) | -1.12 (-.90) | .18 (.31) | .02 (.02) | 112.37 (119.35) | 59 (62) | .00 (.00) |
|  | 23 | .84 (.85) | 2.55 (2.64) | -3.61  (-3.68) | -2.95  (-2.95) | -2.02  (-1.87) | -.87  (-.70) | .50 (.61) | .02 (.02) | 94.10 (106.39) | 62 (67) | .01 (.00) |
|  | 24 | .86 (.87) | 2.76 (2.89) | -3.62  (-3.50) | -2.90  (-2.81) | -2.00  (-1.85) | -.82  (-.66) | .51 (.59) | .02 (.01) | 94.71 (82.22) | 58 (61) | .00 (.04) |
|  | 25 | .84 (.83) | 2.96 (2.96) | -3.58  (-3.47) | -2.83  (-2.76) | -2.05  (-1.97) | -1.03 (-.85) | .22 (.38) | .02 (.02) | 106.68 (82.72) | 55 (58) | .00 (.02) |
|  | 26 | .88 (.87) | 3.58 (3.46) | -3.36  (-3.32) | -2.60  (-2.48) | -1.86  (-1.77) | -.94  (-.80) | .21 (.35) | .02 (.01) | 80.31 (68.31) | 51 (53) | .01 (.08) |
|  | 27 | .85 (.85) | 3.34 (3.31) | -3.62  (-3.20) | -2.61  (-2.54) | -1.82  (-1.66) | -.91  (-.68) | .27 (.47) | .02 (.02) | 92.81 (80.09) | 54 (58) | .00 (.03) |
|  | 28 | .88 (.87) | 3.95 (3.94) | -3.51  (-3.53) | -2.57  (-2.57) | -2.01  (-1.85) | -1.09 (-.91) | .00 (.16) | .03 (.02) | 98.55 (74.46) | 45 (49) | .00 (.01) |
|  | 29 | .83 (.84) | 2.56 (2.79) | -4.00  (-3.68) | -2.71  (-2.67) | -1.96  (-1.87) | -.90  (-.77) | .35 (.43) | .03 (.03) | 138.64 (140.14) | 66 (63) | .00 (.00) |
|  | 30 | .87 (.87) | 3.53 (3.42) | -3.75  (-3.65) | -2.73  (-2.79) | -2.03  (-2.00) | -1.14 (-.98) | .05 (.21) | .01 (.02) | 65.16 (72.99) | 49 (52) | .06 (.03) |
|  | 31 | .86 (.86) | 3.21 (3.17) | -4.07  (-3.55) | -2.69  (-2.72) | -1.90  (-1.84) | -.98  (-.85) | .17 (.31) | .02 (.02) | 96.18 (82.07) | 53 (57) | .00 (.02) |
|  | 32 | .87 (.88) | 3.06 (3.36) | -3.53  (-3.67) | -2.80  (-2.64) | -1.87  (-1.67) | -.82  (-.61) | .50 (.57) | .03 (.01) | 110.93 (80.95) | 55 (60) | .00 (.04) |
|  | 33 | .89 (.91) | 3.53 (3.76) | -3.74  (-3.33) | -2.87  (-2.65) | -1.97  (-1.83) | -.96  (-.71) | .28 (.41) | .03 (.02) | 122.61 (66.69) | 49 (49) | .00 (.05) |
|  | 34 | .89 (.90) | 3.15 (3.32) | -3.66  (-3.46) | -2.74  (-2.50) | -1.92  (-1.71) | -.83  (-.58) | .46 (.59) | .02 (.02) | 77.15 (98.27) | 50 (58) | .01 (.00) |
|  | 35 | .85 (.85) | 2.88 (2.99) | -3.41  (-3.43) | -2.79  (-2.69) | -1.97  (-1.77) | -.96  (-.80) | .25 (.36) | .02 (.01) | 99.64 (77.21) | 60 (60) | .00 (.07) |
|  | 36 | .65 (.71) | 1.47 (1.79) | -3.19  (-3.07) | -2.64  (-2.46) | -1.74  (-1.69) | -.73  (-.62) | .60 (.58) | .02 (.02) | 248.96 (185.43) | 144 (131) | .00 (.00) |

**Supplementary Table 5.** (continued)

| Factor/  Subscale | Item | Factor loadings | IRT item discrimination and difficulty | | | | | | IRT item fit | | | |
| --- | --- | --- | --- | --- | --- | --- | --- | --- | --- | --- | --- | --- |
|  |  |  | a | b1 | b2 | b3 | b4 | b5 | RMSEA | $S\text{-}\chi^{2}$ | $S\text{-}\chi^{2}$ *df* | $S\text{-}\chi^{2}$ *p* |
| Social-Emotional Instructional Strategies  (18 items; Items 36 to 53) | 37 | .83 (.85) | 2.53 (2.75) | -3.19  (-3.03) | -2.39  (-2.29) | -1.61  (-1.47) | -.58  (-.44) | .81 (.81) | .02 (.01) | 130.98 (122.63) | 94 (97) | .01 (.04) |
|  | 38 | .82 (.84) | 2.57 (2.70) | -3.09  (-3.10) | -2.31  (-2.30) | -1.53  (-1.46) | -.55  (-.40) | .87 (.93) | .02 (.02) | 155.93 (132.35) | 99 (97) | .00 (.01) |
|  | 39 | .84 (.85) | 2.93 (2.98) | -3.16  (-2.97) | -2.31  (-2.29) | -1.62  (-1.47) | -.56  (-.38) | .79 (.83) | .01 (.01) | 95.98 (110.52) | 82 (85) | .14 (.03) |
|  | 40 | .84 (.83) | 2.47 (2.57) | -3.63  (-3.47) | -2.84  (-2.70) | -2.09  (-2.00) | -1.13 (-.91) | .22 (.30) | .02 (.02) | 120.95 (144.63) | 82 (91) | .00 (.00) |
|  | 41 | .83 (.86) | 3.02 (3.21) | -3.07  (-3.15) | -2.32  (-2.32) | -1.54  (-1.44) | -.52  (-.37) | .78 (.80) | .01 (.02) | 90.65 (144.73) | 80 (79) | .20 (.00) |
|  | 42 | .88 (.89) | 3.46 (3.57) | -2.99  (-2.98) | -2.50  (-2.46) | -1.73  (-1.59) | -.66  (-.52) | .64 (.66) | .01 (.02) | 81.84 (110.36) | 69 (69) | .14 (.00) |
|  | 43 | .82 (.83) | 2.83 (2.89) | -2.93  (-2.91) | -2.31  (-2.26) | -1.45  (-1.34) | -.46  (-.31) | .80 (.86) | .02 (.02) | 128.62 (142.69) | 89 (93) | .00 (.00) |
|  | 44 | .82 (.84) | 2.79 (3.00) | -3.29  (-3.12) | -2.58  (-2.38) | -1.66  (-1.52) | -.67  (-.49) | .59 (.69) | .01 (.01) | 85.90 (100.01) | 77 (84) | .23 (.11) |
|  | 45 | .86 (.87) | 3.15 (3.35) | -3.35  (-3.39) | -2.55  (-2.41) | -1.74  (-1.53) | -.68  (-.54) | .63 (.65) | .01 (.01) | 79.24 (83.13) | 71 (77) | .24 (.30) |
|  | 46 | .84 (.85) | 2.87 (2.97) | -3.55  (-3.49) | -2.65  (-2.49) | -1.78  (-1.56) | -.76  (-.55) | .59 (.66) | .02 (.01) | 107.03 (98.84) | 76 (80) | .01 (.08) |
|  | 47 | .85 (.86) | 2.89 (3.10) | -3.38  (-3.20) | -2.68  (-2.50) | -1.81  (-1.64) | -.85  (-.66) | .51 (.56) | .02 (.01) | 140.66 (103.17) | 74 (78) | .00 (.03) |
|  | 48 | .83 (.85) | 2.71 (3.07) | -3.33  (-3.18) | -2.61  (-2.43) | -1.60  (-1.46) | -.61  (-.46) | .66 (.69) | .01 (.01) | 87.14 (97.98) | 83 (81) | .36 (.10) |
|  | 49 | .79 (.81) | 2.45 (2.68) | -2.90  (-2.86) | -2.18  (-2.10) | -1.38  (-1.34) | -.44  (-.32) | .84 (.84) | .02 (.01) | 151.07 (138.7) | 111 (105) | .01 (.02) |
|  | 50 | .78 (.80) | 2.24 (2.45) | -3.00  (-3.01) | -2.28  (-2.22) | -1.39  (-1.33) | -.31  (-.27) | 1.10 (.06) | .02 (.02) | 171.92 (147.44) | 111 (107) | .00 (.01) |
|  | 51 | .84 (.87) | 2.73 (3.16) | -3.61  (-3.26) | -2.64  (-2.40) | -1.73  (-1.56) | -.72  (-.53) | .61 (.65) | .02 (.02) | 120.67 (117.22) | 77 (80) | .00 (.00) |
|  | 52 | .86 (.87) | 2.79 (3.10) | -3.42  (-3.28) | -2.61  (-2.41) | -1.70  (-1.50) | -.69  (-.54) | .60 (.64) | .02 (.01) | 114.11 (107.48) | 77 (81) | .00 (.03) |
|  | 53 | .84 (.85) | 2.65 (3.01) | -3.32  (-3.16) | -2.57  (-2.37) | -1.70  (-1.52) | -.60  (-.48) | .68 (.68) | .02 (.01) | 121.56 (109.88) | 80 (83) | .00 (.03) |
|  | 54^♦^ | .89 (.91) | 2.84 (3.05) | -3.52  (-3.25) | -2.54  (-2.41) | -1.75  (-1.55) | -.75  (-.55) | .76 (.78) | .03 (.03) | 49.39 (50.5) | 23 (21) | .00 (.00) |

**Supplementary Table 5.** (continued)

| Factor/  Subscale | Item | Factor loadings | IRT item discrimination and difficulty | | | | | | IRT item fit | | | |
| --- | --- | --- | --- | --- | --- | --- | --- | --- | --- | --- | --- | --- |
|  |  |  | a | b1 | b2 | b3 | b4 | b5 | RMSEA | $S\text{-}\chi^{2}$ | $S\text{-}\chi^{2}$ *df* | $S\text{-}\chi^{2}$ *p* |
| Responses to Challenging Behavior  (5 items; Items 54 to 58) | 55 | .90 (.90) | 4.27 (4.28) | -3.29  (-3.32) | -2.70  (-2.53) | -1.81  (-1.72) | -.89  (-.69) | .36 (.44) | .03 (.03) | 34.40 (39.21) | 14 (14) | .00 (.00) |
|  | 56 | .86 (.88) | 3.86 (4.17) | -3.25  (-3.22) | -2.72  (-2.65) | -1.84  (-1.73) | -.93  (-.71) | .32 (.42) | .03 (.00) | 40.33 (12.59) | 14 (15) | .00 (.63) |
|  | 57 | .89 (.90) | 3.33 (3.51) | -3.47  (-3.37) | -2.76  (-2.58) | -1.88  (-1.73) | -.79  (-.65) | .52 (.59) | .02 (.03) | 23.98 (45.69) | 17 (16) | .12 (.00) |
|  | 58 | .84 (.85) | 3.10 (3.47) | -3.56  (-3.40) | -2.80  (-2.64) | -2.19  (-1.97) | -1.09 (-.89) | .21 (.24) | .04 (.04) | 58.61 (68.15) | 18 (18) | .00 (.00) |
| Interventions for Children with Persistent Challenging Behavior  (5 items; Items 59 to 63) | 59 | .85 (.88) | 2.60 (2.81) | -2.07  (-2.12) | -1.47  (-1.52) | -.83  (-.80) | .08  (.08) | 1.28 (1.13) | .04 (.05) | 169.05 (220.42) | 45 (43) | .00 (.00) |
|  | 60 | .83 (.85) | 5.55 (5.52) | -1.38  (-1.43) | -.91  (-.98) | -.37  (-.43) | .35  (.32) | 1.24 (1.19) | .04 (.04) | 117.39 (113.66) | 32 (30) | .00 (.00) |
|  | 61 | .84 (.83) | 4.90 (4.67) | -1.28  (-1.36) | -.81  (-.90) | -.27  (-.34) | .42  (.44) | 1.39 (1.35) | .04 (.05) | 117.41 (139.12) | 32 (32) | .00 (.00) |
|  | 62 | .95 (.96) | 2.29 (2.59) | -2.48  (-2.28) | -1.68  (-1.63) | -.93  (-.86) | .06  (.11) | 1.36 (1.26) | .05 (.05) | 265.69 (200.00) | 47 (44) | .00 (.00) |
|  | 63 | .89 (.91) | 2.24 (2.54) | -2.06  (-1.97) | -1.44  (-1.40) | -.68  (-.66) | .24  (.25) | 1.53 (1.41) | .03 (.03) | 139.39 (141.11) | 50 (48) | .00 (.00) |
| Supporting Family Use of Social-Emotional Practices  (7 items; Items 64 to 70) | 64 | .94 (.94) | 3.19 (3.33) | -2.56  (-2.44) | -1.91  (-1.82) | -1.20  (-1.09) | -.27  (-.14) | .95 (.94) | .04 (.04) | 193.82 (210.98) | 53 (53) | .00 (.00) |
|  | 65 | .89 (.90) | 3.82 (4.11) | -2.00  (-1.95) | -1.46  (-1.46) | -.89  (-.82) | -.03 (.05) | 1.13 (1.05) | .03 (.04) | 133.00 (174.52) | 54 (50) | .00 (.00) |
|  | 66^*^ | .89 (.89) | 3.94 (4.15) | -1.90  (-1.94) | -1.38  (-1.42) | -.80  (-.78) | .05  (.12) | 1.15 (1.10) | .03 (.03) | 132.85 (124.35) | 50 (49) | .00 (.00) |
|  | 67 | .92 (.93) | 4.40 (4.45) | -2.26  (-2.15) | -1.66  (-1.67) | -1.01  (-.93) | -.11  (-.04) | 1.00 (1.01) | .03 (.03) | 92.82 (107.11) | 41 (43) | .00 (.00) |
|  | 68 | .92 (.92) | 4.09 (4.10) | -2.18  (-2.15) | -1.61  (-1.62) | -1.01  (-.96) | -.16  (-.06) | 1.01 (1.00) | .04 (.03) | 154.18 (124.98) | 46 (45) | .00 (.00) |
|  | 69 | .90 (.90) | 4.11 (4.20) | -1.95  (-1.95) | -1.40  (-1.48) | -.82  (-.79) | .00  (.04) | 1.13 (1.13) | .03 (.03) | 100.35 (98.65) | 50 (49) | .00 (.00) |
|  | 70 | .81 (.82) | 2.74 (2.86) | -1.85  (-1.87) | -1.34  (-1.36) | -.70  (-.70) | .09  (.13) | 1.28 (1.24) | .04 (.05) | 240.78 (284.45) | 68 (66) | .00 (.00) |

*Note.* Standardized factor loadings and IRT parameters for the SETP-C items on the *How Confident* section are presented in parentheses, whereas standardized factor loadings and IRT parameters on the *How Often* section are presented without parent without parentheses.

* represents items flagged for DIF on the *How Often* section, whereas ♦represents items flagged for DIF on the *How Confident* section.

**Supplementary Figure 1.** Conceptual Framework for Developing the SETP-C


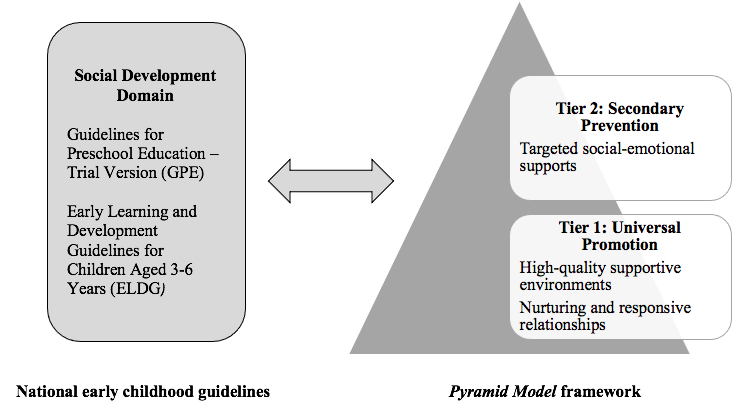


**Supplementary Figure 2.** Four Phases of the SETP-C Development and Content Validation

**Phase 1: Item Generation and Selection**

**Examining alignment** (TPOT, China’s GPE & ELDG)

**Field Observation** (administered TPOT in 20 Chinese preschool classrooms)

**Systematic review** of the Chinese empirical literature (five English and four Chinese databases)

**Item pool** and the preliminary draft of the SETP-C

**Phase 2: Content Validation with Chinese Practice Experts**

**Participants** (205 Chinese practice experts from 31 provinces)

**Measure** (content validation rating scale: *importance* & *cultural relevance*)

**Item reduction procedures** (statistical evidence and clinical considerations)

**Revised version** of the SETP-C

**Phase 3: Content Validation with Chinese Research Experts**

**Participants**

(5 Chinese early childhood professors & researchers)

**Content validation interview** (conceptual basis, proposed measure use, item on the revised version of the SETP-C, additional feedback)

**Recommended revisions**

**Revised version** of the SETP-C

**Phase 4: Response Process Validation Using Cognitive Interviews**

**Participants**

(5 in-service and 5 pre-service Chinese preschool teachers)

**Cognitive interviewing** (think-aloud & verbal probing)

**Written feedback** (any concerns about the wording and translation)

**Final version** of the SETP-C
